# Aging, Maturation and Growth of Sauropodomorph Dinosaurs as Deduced from Growth Curves Using Long Bone Histological Data: An Assessment of Methodological Constraints and Solutions

**DOI:** 10.1371/journal.pone.0067012

**Published:** 2013-06-19

**Authors:** Eva Maria Griebeler, Nicole Klein, P. Martin Sander

**Affiliations:** 1 Department of Ecology, Zoological Institute, University of Mainz, Mainz, Germany; 2 Steinmann Institute, Division of Paleontology, University of Bonn, Bonn, Germany; Ludwig-Maximilians-Universität München, Germany

## Abstract

Information on aging, maturation, and growth is important for understanding life histories of organisms. In extinct dinosaurs, such information can be derived from the histological growth record preserved in the mid-shaft cortex of long bones. Here, we construct growth models to estimate ages at death, ages at sexual maturity, ages at which individuals were fully-grown, and maximum growth rates from the growth record preserved in long bones of six sauropod dinosaur individuals (one indeterminate mamenchisaurid, two *Apatosaurus* sp., two indeterminate diplodocids, and one *Camarasaurus* sp.) and one basal sauropodomorph dinosaur individual (*Plateosaurus engelhardti*). Using these estimates, we establish allometries between body mass and each of these traits and compare these to extant taxa. Growth models considered for each dinosaur individual were the von Bertalanffy model, the Gompertz model, and the logistic model (LGM), all of which have inherently fixed inflection points, and the Chapman-Richards model in which the point is not fixed. We use the arithmetic mean of the age at the inflection point and of the age at which 90% of asymptotic mass is reached to assess respectively the age at sexual maturity or the age at onset of reproduction, because unambiguous indicators of maturity in Sauropodomorpha are lacking. According to an AIC-based model selection process, the LGM was the best model for our sauropodomorph sample. Allometries established are consistent with literature data on other Sauropodomorpha. All Sauropodomorpha reached full size within a time span similar to scaled-up modern mammalian megaherbivores and had similar maximum growth rates to scaled-up modern megaherbivores and ratites, but growth rates of Sauropodomorpha were lower than of an average mammal. Sauropodomorph ages at death probably were lower than that of average scaled-up ratites and megaherbivores. Sauropodomorpha were older at maturation than scaled-up ratites and average mammals, but younger than scaled-up megaherbivores.

## Introduction

Life history traits such as life span, age at sexual maturity, and maximum growth rate are important factors for understanding the biology of any organism, but they are most important in a comparative context in studies of evolution and evolutionary ecology. This is because certain kinds of trade-offs such as investment in individual growth versus maturation underlie the evolutionary structure of the life history traits of an organism.

Although there is an ongoing debate about whether the scaling of life history traits follows a universal power law [1]–[3], it is generally accepted that such traits in extant taxa follow allometries [4]. Allometries are relationships between one trait (*x*) and another trait (*y*) of an organism compared to organisms of other species or to individuals of the same species but of another ontogenetic stage. They are often mathematically described by a power function *y* = *a* exp(*b*) [5]–[7], and thus in a log-log plot allometries are linear functions *y a*+*b*⋅*x* to *y=a*+*b*⋅*x*. When trait *y* scales with trait *x* and the exponent *b* is one, the allometry is called an isometry. Allometries between body mass (*x*) and any other trait (*y*) estimated for a taxon (e.g., extant non-avian reptiles) are often used to estimate trait *y* for another taxon, e.g., to estimate the maximum growth rate of an extinct dinosaur from its body mass. The scarcity of estimates of life history traits has so far limited the detection of reliable allometries between body mass and life history traits for dinosaurs, and in particular for Sauropodomorpha, which include the largest terrestrial animals that ever lived on Earth, the Sauropoda [8], [9]. In spite of this scarcity, presumed differences in the life history of sauropods in comparison to extant taxa have been suggested to contribute to their unique gigantism [9]–[13].

Whereas in extant organisms biologists can directly observe variability in life history traits in the taxa on which the allometries are based, this is often not possible in extinct organisms as traits are simply not preserved in the fossil record (e.g., number of broods per year, age-specific mortality rates). However, for complete tests of hypotheses in evolutionary biology and evolutionary ecology, we need to include fossil organisms. Fossil organisms reflect the phylogenetic history and the ancient states of traits of extant species.

In dinosaurs, besides the discovery of fossil eggs and clutches, the most important source of information on their life history traits is the growth record preserved in the histology of their fossilized bones. It is the aim of this study to provide, for the first time, baseline data on allometries of aging, maturation, and growth for Sauropodomorpha. To obtain these allometries between body mass and life history traits, we studied the long bone histology of a single individual of the basal sauropodomorph *Plateosaurus engelhardti* and of six sauropod individuals pertaining to four different taxa, which have a good growth mark record preserved in their humerus or femur (Table 1). On the basis of growth marks, we establish individual growth curves by applying and testing the following sigmoidal growth models [14]: von Bertalanffy (vBGM), Gompertz (GGM), logistic (LGM), and Chapman-Richards (CRGM). These growth models have been successfully applied to extant species (and some also to dinosaurs as reviewed below), but have been rarely statistically tested against each other for a specimen (but see Cooper et al. [15] who considered five different growth models) and checked for biological reliability. For each of the individuals, we estimated four life history traits, that is, “age at death”, “age at which the individual is fully-grown”, “age at sexual maturity”, and “maximum growth rate” from the growth curves. We then used these estimates to establish allometries between body mass and each of the life history traits. We are aware that the uncertainties involved in establishing these allometries are much greater than in an extant dataset. We, nevertheless, feel our approach is justified because of the important insights to be gained from this work, by making a margin of error much greater than the customarily acceptable 10%. To validate our allometric hypotheses for populations and even species, and their applicability to Sauropodomorpha in general, more individuals have to be tested.

**Table 1:** Data basis of the current study.

Taxon (Locality)	Spec. No.	Bone and length (mm)	Length of section (mm)	Number of cycles	Body mass (kg)	Specific density	HOS	Bone tissue types
*Plateosaurus engelhardti* (Tr)	IFG 192.1	Femur (740)	28	12^+^ (min = 3, max = 9)	1,587	1.0	5–10	D–F
indet. mamenchisaurid (SF)	SGP 2006/9	Humerus (1400)*	76	15 (+6 EFS) (min = 3.5, max = 9)	25,075	0.9	12	F - EFS
*Apatosaurus* sp. (MF)	SMA 0014	Femur (1640)	74	20 (min = 2, max. = 7–8)	20,206	0.9	10	E–F
*Apatosaurus* sp. (MF)	BYU 601–17328	Femur (1580)	58	5 (+13 EFS) (min = 11, max = 13)	18,178	0.9	12	F - EFS
indet. diplodocid (TB)	MfN.R.2625	Humerus (610)	40	9 (min. 8–9, max = 15)	4,144	0.9	10	E–F
indet. diplodocid (TB)	MfN.R.NW4	Femur (1350)	62	16 (min = 2, max = 7)	11,632	0.9	5–11	B–F
*Camarasaurus* sp. (MF)	CM 36664	Femur (1450)	79	5 (+4 EFS) (min = 7–8, max = 17)	14,247	0.9	12	F - EFS

### The growth record preserved in sauropodomorph long bones

In dinosaurs, and especially sauropodomorphs, bone histology has mainly focused on the mid-shaft cortex of the humerus and femur because a good record of appositional growth is preserved [16]. Histology of sauropodomorph long bones is rather uniform, consisting of a thick cortex of primary bone most of which is of the laminar fibrolamellar kind [17]. Laminar fibrolamellar bone is a fast growing bone tissue that consists of a framework of woven-fibered bone tissue with mainly circumferentially arranged primary osteons giving the tissue its laminar appearance [17]. In fully-grown individuals, the outermost cortex consists of a very thin layer of avascular parallel-fibered or lamellar bone with closely spaced lines of arrested growth. This layer is called an external fundamental system (EFS), and in animals such as dinosaurs and large mammals growing with fibrolamellar bone throughout most of their life, the EFS is very distinctive and records a very sudden decrease in growth (see Sander et al. [17]).

A quantifiable growth mark record throughout ontogeny, which is potentially informative about the timing of important life history events and the increase in body mass, is present only in a minority of sauropod humeri and femora [16], [17]. This is contrary to the widespread growth mark record in Ornithopoda and Theropoda (e.g., [18]–[22]) and in basal Sauropodomorpha [23]–[25], as exemplified by the *Plateosaurus* specimen in this study. Among the more than 250 sauropod specimens examined by us over the years [16], [17], only a limited number of samples preserve a growth mark record sufficient for quantification of individual growth. In the few cases where growth marks are deposited in sauropods they occur only late in ontogeny and most often only an EFS is deposited in the outermost cortex [16].

Reasons for the poor growth mark record are twofold: remodeling/resorption processes and non-deposition. The inner part of the growth mark record is destroyed by resorption through enlargement of the medullary cavity and by remodeling of the bone. In primary bone lacking growth marks, growth may have been too fast for growth mark deposition [16], [17]. The annual cyclicity of growth marks is generally accepted for dinosaurs [17], [20], [26] and is supported by the most recent study of growth marks in extant animals with a very similar histology to that of dinosaurs, ungulate mammals. Köhler et al. [27] unequivocally and overwhelmingly documented the annual nature of the growth marks in ungulates, regardless of habitat, climate, and latitude.

Certain methodological problems occur, however, when counting growth marks in sauropod dinosaur bone. Because growth marks are absent in the inner part of the bone, the number of growth marks preserved is only the minimal age at death of the individual. While the annual nature of growth marks in fibrolamellar bone tissue is unequivocal, those in the EFS, recording the asymptotic phase of growth, may not (all) be annual. Finally, the histology of primary bone no longer records life history once growth has ceased completely. Thus, extant crocodiles can live for many years after reaching full size [22], [28], and the same may well have been true for Sauropodomorpha.

Long bone histology is informative about body mass increase because the dimensions of long bones are the best proxies of body mass [9], [17], [29]. For calculating body mass increase from growth marks, the observation that both humerus and femur diameter scale isometrically with the length of these bones [30], [31] is very helpful. Because bone length isometrically scales with body mass, the isometric length to diameter scaling leads in turn to a close isometric relationship between the increase in cortex thickness and body mass [17].

### Previous studies of sauropod growth curves and growth rates

Erickson et al. [32] were the first to quantify growth rates for a group of dinosaurs, spanning phylogenetic and size diversity. Among those data was also a multi-individual growth curve for *Apatosaurus*, which extrapolates an age of 15 years at asymptotic mass. Initial doubts about this growth curve and the resulting growth rates for *Apatosaurus* were raised by the description of an *Apatosaurus* specimen by Sander and Tückmantel [33] that preserved 26 growth cycles only in its outermost cortex. Other studies also came to the conclusion that the maximum growth rate estimates of Erickson et al. [32] for *Apatosaurus* represent a clear overestimate ([17], [34], this study). Lehman and Woodward [34] noted that the reason for this overestimate was the impossibility of counting growth lines in *Apatosaurus* bones larger than about two-thirds adult length because secondary bone remodeling had obliterated the primary bone tissue.

The only other study to provide growth curves for sauropods is that by Lehman and Woodward [34], which dealt with four different taxa. While offering a convincing methodology for curve fitting, for three taxa (*Apatosaurus*, *Janenschia*, indeterminate sauropod from England) out of the four investigated, the study suffers from different problematic assumptions. We suggest that only the growth curve for *Alamosaurus* can be considered reliable.

Lehman and Woodward [34] reanalyzed the *Apatosaurus* growth series published by Curry [35]. Their *Apatosaurus* growth curve, however, is poorly constrained because it is based on the scapular growth record. Only large limb bones have been proven to have a close isometric relationship between bone circumference and length [30], [31], while the isometry of the shape of the sauropod scapula remains untested. Thus, we do not know how the increase in cortical thickness of the medial surface of the shaft of the scapula translates into increasing body mass. Also, it is has not been tested if growth of the humerus and femur is comparable to that of the flat and more curved scapula, because different elements of the skeleton show different growth patterns [18], [36], and therefore may preserve different growth records as was also shown for *Plateosaurus* [25].

The growth record underlying the growth curve of the indeterminate sauropod from England [34] was based on published information on the pubis of a single individual [37]. We rate the resulting growth curve problematic for similar reasons as for the scapula of *Apatosaurus*. Additionally, the body mass of any sauropod cannot be reliably estimated from the dimensions of its pubis.

The growth curve for the poorly known macronarian sauropod *Janenschia* was fitted by Lehman and Woodward [34] based on the information and illustrations published by Sander [38] for a single femur of this taxon combined with a poorly constrained estimate of asymptotic mass [17]. Because there were major differences between the growth record used by Lehman and Woodward [34] and the original record used by us, we did not expect to corroborate their growth model. We finally excluded *Janenschia* from our study, because none of the four tested growth models (vBGM, GGM, LGM, CRGM) revealed a reliable growth curve.

In contrast, the growth curve for the derived titanosaur *Alamosaurus sanjuanensis* from Lehman and Woodward [34] is the most reliable growth curve for a sauropod published so far. We consider this multi-individual growth curve for *Alamosaurus sanjuanensis* well constrained for several reasons: First, this taxon is known from entire skeletons, making a reliable mass estimate possible. Second, at least some bones preserve a good histological growth record. Third, the data are original to the study of Lehman and Woodward [34].

### Problems of existing approaches to growth curve fitting in dinosaurs

As a consequence of the incompletely preserved growth record in dinosaur long bones, two standard sigmoidal growth models [14], the vBGM [34] and the LGM [19], [32], [39], have mostly been applied to dinosaurs, but authors have rarely tested different standard growth models for one growth record against each other (but see Cooper et al. [15] who considered five different growth models). Different regression approaches have also been used. Erickson et al. [19], [32] and Bybee et al. [39] estimated the initial body mass, the final body mass and the growth rate of the logistic growth curve by non-linear regression analysis, although growth marks were scarce in comparison to the number of estimated model parameters. Lehman and Woodward [34] overcame this problem by estimating only the growth rate of the curve and setting the initial body mass to zero and the final body mass to the body mass of the largest known individual of that taxon.

However, the standard models used so far for assessing growth curves in dinosaurs inherently make assumptions that may be problematic when inferring information on life history traits from the growth curve. Estimates obtained for age at death of the individual, age at sexual maturity, and the age at which the individual is fully-grown (hereafter asymptotic age of the individual) are generally biased by the unknown number of growth cycles that are not preserved in the bone and estimates of age at death are additionally biased by the number of years of life after growth ceased completely. The inflection point in a growth curve, which marks the transition from growth acceleration to deceleration, has been used to estimate age at sexual maturity of dinosaurs [34], [40]. Unambiguous indicators of sexual maturity in fossils are rare (for fossil evidence in other dinosaurs see Lee and Werning [40] and Erickson et al. [41]) and especially are lacking in sauropodomorph dinosaurs. From a phylogenetic perspective, sexual maturity around the inflection point is the generalized condition for amniotes, and it is most parsimonious to assume that this applied to dinosaurs as well [38], [40], [41]. However, the standard growth models such as the vBGM and the LGM have a fixed inflection point at about 30% and 50% of asymptotic mass, respectively [14]. Because most authors [19], [32], [39], [41]–[43] had only applied these two standard growth models to dinosaur growth records, they *a priori* fixed the age at which growth starts to decelerate rather than estimated it. More flexible growth models that explicitly parameterize the position of the inflection point in the growth curve, such as the CRGM [44], are more appropriate for estimating the age at which growth of an individual starts to decelerate than the standard growth models that have been applied so far [15]. The CRGM has been successfully applied to various extant mammals [45].

The concept of the inflection point of the growth curve indicating sexual maturity requires, however, further comments. Theoretical studies [46], [47] have suggested that the inflection point reflects the beginning of the individual's investment in reproduction at the expense of future somatic growth. Empirical studies, however, often demonstrate an onset of reproduction at ages well past the inflection point and thus suggest that this point should be better interpreted as the lower limit of reproductive maturity. Nevertheless, evidence for the concept of the inflection point indicating the beginning of reproduction exists, e.g. in reptiles [40], [48] and in large mammals such as female elephants [40], [45]. However, in smaller mammals [40], the first reproduction is observed when animals are more or less fully-grown, and in birds [41], [49] reproduction starts close to the attainment of final size and is thus mostly delayed compared to the age at which the inflection point of the growth curve is observed. Factors explaining the beginning of reproduction past the inflection point of the growth curve are the nutritional state of the animal [48], its breeding system (e.g. polygyny, [50], [51]), and seasonal reproduction (especially in species that are fully-grown within their first year of life).

Because the onset of reproduction shows such high variability in extant taxa, we developed a new bracketing approach in this paper to assess sexual maturity from individual growth curves. If the earliest possible age at sexual maturity (in the sense of the first reproduction) is the inflection point (lower limit), the latest is the end of the quasi-linear phase of growth (around the inflection point) that is the age at which 90% of the asymptotic body mass is reached (upper limit). The choice of 90% as the upper limit is guided by extant birds reaching reproductive maturity at this time ([49], [52]), giving us the worst case known in extant species as the possibly latest time of sexual maturity in our bracket. We then take the arithmetic mean of the age at the inflection point and the age at 90% asymptotic body mass as the most likely age at sexual maturity (hereafter *ASM*).

### Histologic ontogenetic stages in sauropodomorph dinosaurs

To qualitatively describe the ontogenetic status of sauropodomorph individuals based on their long bone histology, we earlier had developed the concept of histological ontogenetic stages for Sauropoda (HOS, [16]), which can also be applied to some extent to *Plateosaurus*. We used the sequence pattern of six bone tissue types (A through G) to define 13 HOS and hypothesized that sexual maturity coincides with the onset of bone tissue type E deposition. We also correlated the 13 HOS with seven biological ontogenetic stages (embryo, hatchling, juvenile, subadult, adult I = significant growth after sexual maturity, adult II = growth slows significantly down; adult III = no growth; plus substages) and to specific life history events (hatching, sexual maturity, senescence). Sexual maturity is correlated with HOS 8. Thus, the HOS concept can be used to assess the biological plausibility of growth curves fitted to the growth record of Sauropodomorpha.

## Materials and Methods

### Histological sampling and sample base

This study is based on seven long bones (Table 1) from five sauropodomorph taxa (*Plateosaurus engelhardti*, a giant mamenchisaurid, *Apatosaurus* sp., Diplodocinae indet., *Camarasaurus* sp.), each bone representing one individual. The samples are part of a large histological collection of sauropodomorph long bones [16], [17], [25] that includes thin sections and polished sections. The current study is mainly based on polished sections, which sometimes tend to show subtle growth marks better than thin sections. Table 1 provides the database for the study and lists all pertinent information such as taxa, specimens, geologic age, localities, length of the section (as a proxy for thickness of the cortex) and calculated mass of individuals, number of visible growth marks, and histological ontogenetic stages preserved in the bone. The sections were chosen for this study because they are the only ones showing reliable quantifiable growth marks in their cortex and the only ones for which we have reliable body mass estimates of the individuals sampled.

None of the samples are diagnostic to species because they came from isolated long bones, which in sauropods are at best diagnostic to genus. Thus, the long bones assigned to *Barosaurus africanus* by Janensch [53] and identified as such in previous histological studies [16], [33], [38] were more recently found to be undiagnostic beyond Diplodocidae [54]. However, we note that these taxonomic uncertainties are irrelevant to the major conclusions of this study.

All bones, except for the indeterminate mamenchisaurid humerus, were sampled by core drilling with a diamond drill bit (diameter of about 13 mm or 16 mm) at a standardized sampling location in the midshaft region of the bones. This is the posterior side in the humeri and the anterior side of the femora, as described by Sander [38] and Stein and Sander [55]. The cores were embedded in epoxy resin and cut perpendicular to the long axis of the bone. The cores were then processed into polished sections and thin sections by standard petrographic methods. The indeterminate mamenchisaurid humerus was sampled by sectioning across the entire midshaft, and only a polished section was prepared. In contrast to the core-sampled humeri, the growth record of this specimen was obtained from the anterior bone side. We are aware that distances between growth marks can vary along the bone circumference, and that this could eventually bias estimates of life history traits derived from fitted growth curves. Nevertheless, as the data base for growth curves is limited in Sauropodomorpha, we think this was justified.

**Figure 1:**
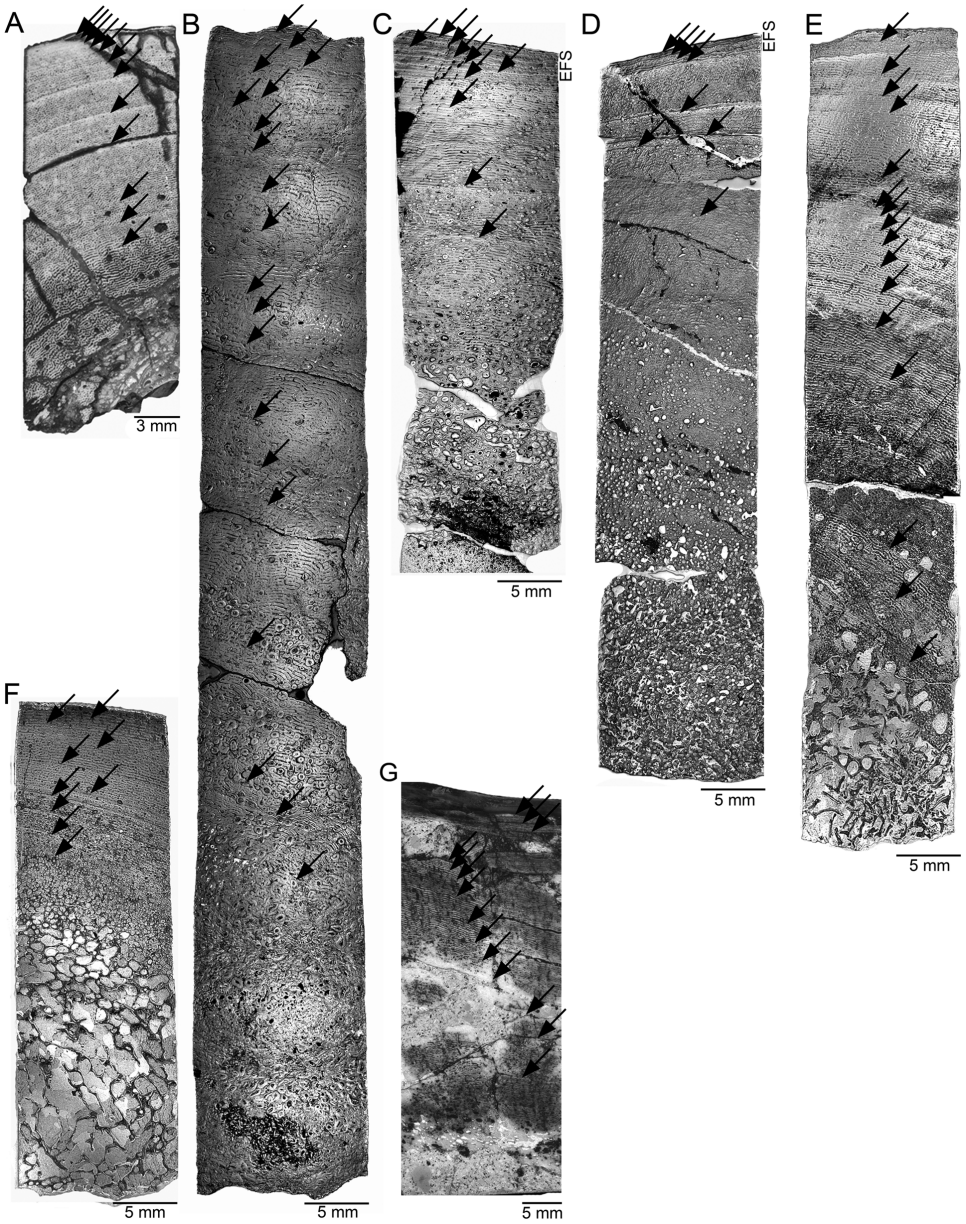
Growth mark record in polished sections of sauropodomorph individuals used in this study. Arrows indicate considered growth marks which are oriented parallel to the growth direction of the bone surface. The outer bone surface is at the top. Most samples extend from the outer cortex into the medullary region, but the center of medullary region is not covered by all samples. The primary bone tissue generally is laminar fibrolamellar bone. All samples except for the indeterminate mamenchisaurid sample are polished sections, which originate from core samples. Except for A) and G), which are photographed in normal incident light, all other images are in bright field illumination, with light reflected off the polished sample surface. **A)** Femur sample of *Plateosaurus engelhardti* (IFG 192.1; 740 mm) with 12 lines of arrested growth deposited. The outermost cortex does not show an external fundamental system (EFS), which indicates that this individual had not yet reached asymptotic growth, but growth rate clearly had decreased due to the close spacing of the growth marks in the outer cortex. **B)** Femur sample of *Apatosaurus* sp. (SMA 0014; 1,640 mm) with 20 growth marks consisting of lines of arrested growth and polish lines. The outer cortex is incomplete due to post mortem damage. **C)** Femur sample of *Apatosaurus* sp. (BYU 601-17328; 1,580 mm) with five growth marks consisting of polish lines. The outer cortex exhibits an EFS which contains another 13 resting lines deposited. **D)** Femur sample of *Camarasaurus* sp. (CM 36664; 1,452 mm) with five growth marks consisting of polish lines and lines of arrested growth. The outermost cortex exhibits an EFS, which contains another four resting lines. **E)** Femur sample of an indeterminate diplodocid (MfN.R.NW4; 1,350 mm) with 16 growth marks in form of lines of arrested growth and polish lines. The outermost cortex lacks an EFS. **F)** Humerus sample of an indeterminate diplodocid (MfN.R.2625, 610 mm) with nine growth marks in the form of lines of arrested growth and polish lines visible in the cortex. The outermost cortex lacks an EFS. **G)** Humerus sample of the indeterminate mamenchisaurid (SGP 2006/9; approx. 1,400 mm) showing 16 growth marks consisting of lines of arrested growth in the outer cortex and cyclic variation in vascularity (modulations) in the inner cortex. The outermost cortex shows an EFS which is too thin to see at the magnification of this image.

Polished sections were examined with a Leica DMLP compound microscope (1.6× to 40× magnification of the objective lenses) in reflected light bright field illumination and with a binocular microscope. Growth marks were counted and recorded in the polished sections either with a binocular microscope with a camera lucida attached in normal light (Figure 1) or on high-resolution digital images of the sections using reflected light (Figure 1). Thin sections were examined by standard light microscopic techniques (normal transmitted light, polarized light) with a Leica DMLP compound microscope (1.6× to 40.0× magnification of the objective lenses).

### Quantification of the growth mark record

To understand the problems involved in quantifying the growth mark record, including the reconstructing of the missing growth record of the inner cortex, a clear understanding of the origin and the nature of this growth record is needed. The shaft of a sauropod humerus or femur grew from hatchling to a fully-grown animal by the periosteal apposition of new bone material, leaving the postnatal growth record behind. Considering that sauropod hatchlings were very small, [56], the beginning of this growth record is close to the geometric center of the shaft cross section, and it ends at the bone surface. However, the growth record is asymmetrical since the apposition rates differed locally, along the circumference of the bone shaft, due to biomechanics.

The older parts of the growth record were destroyed by the expanding medullary region and by remodeling of the inner cortex. Due to the commonly crushed medullary region in sauropod bones, it was not possible to fully reconstruct the original growth record from the geometric center of the medullary cavity to the bone surface. Thus, the length of each thin section as measured from the deepest part to the outer bone surface was set to 100% of the growth record, being well aware that this is only the minimum of cortical apposition for the respective specimen. Distances between cycles and the length of the thin section were measured parallel to the growth direction of the bone surface, because the center of the medullary region is not covered by all samples (Figure 1, direction of arrows).

In most samples, the growth marks do not start before the second half of the section because the inner cortex was heavily remodelled or no growth marks had formed. We therefore estimated a minimum and maximum number of missing growth marks for all specimens by retrocalculations. This information was needed for curve fitting (see below). The minimum number of missing growth marks was assessed by dividing the length of the inner part of the section without growth cycles by the width of the thickest growth cycle. The maximum number of missing growth cycles was estimated by dividing the length of the inner part of the section without growth marks by the average cycle width. Growth marks in the outermost cortex that are closely spaced in avascular bone (EFS) were not included in the calculation of the maximum and minimum number of missing growth cycles.

### Histology and growth record

#### Basal Sauropodomorpha

*Plateosaurus engelhardti* differs from the Sauropoda in that it shows plesiomorphically high developmental plasticity [24] with widely varying final sizes at a given age. From the database of Klein and Sander [25], a femur of 740 mm length (IFG 192.1) of *Plateosaurus engelhardti* was selected for the current study because it shows a relatively complete growth record of a large individual (Figure 1A). The growth record of this specimen preserves bone tissue comparable to that defined for Sauropoda as types D to F of Klein and Sander [16] (except for the remodeling by secondary osteons; [16]) in sequence and thus includes the life history event of sexual maturity (onset of deposition of type E tissue). Towards the outer cortex, a decrease in vascular density is obvious and growth continued at a slower rate but no EFS had been deposited yet. Klein and Sander ([25]: Table 4) estimated that at least three growth cycles are missing in this specimen, resulting in a total estimated number of 15 growth cycles (Figure 1A, Table 1). Supplementary retrocalculations carried out for curve fitting suggest that at most nine cycles are missing.

#### Sauropoda

Unlike *Plateosaurus*, members of Sauropoda show little developmental plasticity, generally exhibiting a close correlation between ontogenetic stage and size [16], [17]. For this reason, we assume that the individuals in this study are representative for their species. The humerus of the indeterminate mamenchisaurid (SGP 2006/9; estimated length 1,400 mm, polished section from the anterior bone side) chosen for this study has so far not been histologically studied and represents one of the largest indeterminate mamenchisaurid sauropod individuals known [57]. The large size of this individual is consistent with the bone histology, which indicates HOS 12 of Klein and Sander [16]. The outermost cortex shows a thin EFS, and little remodeling in the cortical bone, leading to the preservation of a good growth record. Unusually for sauropods, the growth cycles are present throughout most of the cortex, delimited by lines of arrested growth in the outer cortex and cyclical modulations of vascularization in the inner cortex. Although still primary bone, the innermost cortex lacks growth marks (Figure 1G). A total of 16 growth cycles are preserved, and retrocalculation suggests that between 3.5 and 9 cycles are missing, not having been expressed or having been lost due to medullary expansion.

Two femora of *Apatosaurus* sp. were selected from our sample base [16], [17] for the current study because they show growth marks in their cortex (Table 1, Figure 1B, C). SMA 0014 (1,640 mm, sample from the anterior bone side) is a femur and its bone tissue records HOS 10. The length of this femur is 91% of the maximum size that is found in the sample of Klein and Sander [16] for *Apatosaurus*. It has 20 growth cycles visible in its cortex and no EFS had developed yet but the bone surface is incomplete (Figure 1B). A maximum of seven missing growth cycles was previously retrocalculated for this sample, resulting in an age estimate of 28 years at death [17]. The minimum number of missing growth marks estimated for this study is two (Table 1). BYU 601-17328 (1,580 ¸ sample from the anterior side) is a femur of 88% of the maximum size in the *Apatosaurus* sample of Klein and Sander [16]. Although somewhat smaller when compared to SMA 0014, its bone tissue records clearly a later ontogenetic stage (HOS 12, [16]). The section has five growth marks in its cortex and another 13 in an EFS in the outermost cortex (Figure 1C). Retrocalculations suggest that at least 11 and at most 13 growth cycles are missing from the inner part of the bone. The differences in femur length and ontogenetic stages between the two *Apatosaurus* sp. specimens might indicate that these do not belong to the same taxon. However, in most histological work, our null hypothesis is that the identifications found in the collections are correct. Only if there are inconsistencies in, say, an ontogenetic series, do we question the morphological data and suspect a cryptic diversity at the population or species level.

One femur of an indeterminate diplodocid (MfN.R.NW4; 1,350 mm, sample from the anterior bone side) was selected. This femur is 89 % of the maximum known size for Tendaguru diplodocids (Table 1; Figure 1E). It had 16 growth cycles preserved, and between two and seven cycles are missing. This sample of HOS 11 is quite interesting because it shows nearly the entire ontogenetic sequence of bone tissues from type B to type F [16], and thus in particular probable sexual maturity (onset of deposition of type E tissue) is documented in the bone. The second sample of the indeterminate diplodocid showing a good growth record is a humerus (MfN.R.2625; 610 mm, sample from the posterior bone side) that is 59 % of the maximum known size (Table 1; Figure 1F). It preserves nine growth cycles and already shows HOS 10 despite its relative small size. Retrocalculation suggest that between eight and 15 growth cycles are missing.

Of the *Camarasaurus* sp. sample base of Klein and Sander [16], one femur (CM 36664; 1,452 mm, sample from the anterior bone side) was selected that is 93% of the maximum size known for *Camarasaurus* in the sample of Klein and Sander [16] (Table 1; Figure 1D). It records five growth cycles in the outer cortex. The outermost cortex shows an EFS with another four growth marks developed, and the HOS is 12 but with an untypically low vascularization throughout the entire section. Retrocalculation suggests that between seven and 17 growth cycles are missing.

### Mass estimates

To construct a mass-based growth curve for a single individual, the relationship between local bone apposition rate and body mass gain needs to be understood. Because in sauropod long bones, minimal shaft circumference increases proportionally with bone length [30], [31], the local bone apposition rate is closely tied to body mass gain. Using a body mass estimate for the individual at the time at death (*body mass at death* in equation (1)), a growth curve can be constructed by measuring the distances between growth marks, calculating which percentage of size at death was reached at each growth mark, and then transforming this to percentage of body mass at death at each growth mark. Specifically, estimates of mass at each growth mark were calculated following Erickson and Tumanova [42] as
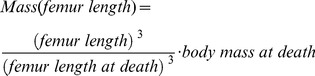
(1)


*Body mass at death* for the two *Apatosaurus* individuals, the two indeterminate Tendaguru diplodocids, and *Camarasaurus* was calculated from the femur length/body mass regression (equation 2 in Table II from Mazzetta et al. [58]: log (body mass) = 2.838 log (femur length) – 4.864); Note: the equation given in the footnote of Table II of Mazzetta et al. has a typographical error), but was corrected for an avian body density of 0.9 because of the very likely presence of an extensive air-sac system in sauropods [59]. Because the indeterminate Tendaguru diplodocid MfN.R.2625 is a humerus, femur length had to be estimated for the body mass estimate. For this, we used a humerus/femur ratio of 0.65 [60]. For *Plateosaurus*, we used the mammalian megaherbivore body density of 1. *Body mass at death* in equation (1) is the estimate from Klein and Sander [25], because no evidence exists for an extensive air-sac system in *Plateosaurus* [61]. Body mass at death for the indeterminate mamenchisaurid individual was 25,075 kg (unpublished data, revised estimate of the value of 31,000 kg published by Sander et al. [17] and Wings et al. [62]).

*Femur length at death* in equation (1) is the length of the studied femur. *Femur length* in equation (1) corresponds to length of the bone at a specific growth mark and was derived from the percentage of the length of the thin section at death.

### Model fitting

To find the best growth model for *Plateosaurus engelhardti* and the six sauropods, we considered the four mathematical growth models mentioned above: von Bertalanffy (vBGM), Gompertz (GGM), logistic (LGM), and Chapman-Richards (CRGM). All relate the mass of an individual *M(t)* to its age *t*. In all models *M_0_* is a (non-zero) initial body mass, *A* is the asymptotic mass of the individual, and *g* is the growth rate for each individual.

#### Von Bertalanffy growth model

The von Bertalanffy growth model [63], [64] is a standard sigmoidal growth model known to visualize increase in body mass of birds [49]. It has also been successfully applied to many extant reptile taxa [65] including snakes, lizards [66], turtles [67], crocodiles [68], and even extinct sauropod dinosaurs [34]. The specific form of the vBGM that we fitted to the histological growth records of Sauropodomorpha is defined as
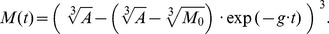
(2)


It is based on the Pütter-von Bertalanffy equation [63], [64], [69]. The inflection point of this growth curve is found at about 30% ( = 100⋅8/27, [14]) of asymptotic mass.

#### Logistic growth model

Logistic growth models have been utilized to describe growth in extant eutherian mammals [45] but also in smaller reptiles [68] including tortoises [48] and extinct dinosaurs [32]. The specific LGM we tested for each of the Sauropodomorpha studied here is
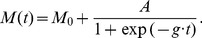
(3)


This growth model assumes a symmetric inflection point at 50% of asymptotic mass [14].

#### Gompertz growth model

The Gompertz model is a third standard growth model that has long been considered as the general model describing mammalian growth [45], [70] and has also been suggested for chelonians [71], [72]. In this model, the inflection point is located at about 37% ( = 100/*e*, [14]) of asymptotic mass. The GGM, however, was not applicable to any of the individuals studied in this paper. The curve fitting procedure always failed, because either the fitting algorithm revealed no parameter estimates or the fitted regression functions were biologically unrealistic (see below). We thus omit the GGM in the remaining parts of the Methods and Results sections.

#### Chapman-Richards growth model

For all sauropodomorph individuals, we also considered a flexible growth model with a variable inflection point that had been successfully applied to modeling growth in mammals [45]. This is the Chapman-Richards growth model that is formulated as
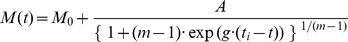
(4)


Parameter *m* sets the inflection point on the body mass axis. Parameter *t_i_* is the age at which the inflection point occurs. Our CRGM is based on the general Chapman-Richards model [44], but we slightly modified this model to allow for non-zero initial body masses. The CRGM is able to generate any sigmoidal growth curve within the two extremes, a monotonic concave increase (no inflection point, maximum growth rate at birth) and a monotonic convex increase (no inflection point, truncated exponential model). By choosing specific values for parameter *m*, the flexible CRGM is able to mimic the vBGM (*m* = 2/3), the GGM (*m*→1, note that equation 4 is not defined for *m* = 1), and the LGM (*m* = 2) [39], [44], [45].

### Numerical model solving

We applied non-linear regression analysis for estimating the parameters of growth models for our sample. Each of the three growth models (equations 2 through 4, vBGM, LGM and CRGM) was tested for each of the seven specimens. The GGM was not applicable to any of the specimens tested here, as noted above. To cope with the problem that an unknown number of cycles are missing from the inner part of the bone due to resorption, remodeling, and non-expression of inner growth marks (due to very fast growth rates), we developed two new numerical curve-fitting techniques and applied these to each of the three growth models.

#### Fitting technique 1 (FT1)

Setting the mass estimate for the first visible growth mark as the initial body mass (*t* = 0) in the growth series and assuming that the sauropod hatchling mass was about 2 kg [56], [73] allowed us to implicitly estimate the number of cycles missing from the inner part of the bone. From the fitted growth curve, we therefore read off the *t* value for which the curve predicted a mass of 2 kg. Because this *t* value is negative (because the mass at the first growth mark is larger than 2 kg), the number of missing cycles is the absolute value of *t* with *M*(*t*) = 2 kg.

#### Fitting technique 2 (FT2)

We set the mass at cycle zero to a fixed hatchling weight of 2 in the growth series and stepwise, considered different initial numbers of missing cycles during the overall curve fitting process. The initial numbers that we manually tested one after another ranged from the minimum to the maximum number of missing growth cycles that we had previously estimated for each of the specimens using retrocalculation methods (Table 1). For example, for *Apatosaurus* sp. (femur BYU 601-17328), from 11 up to 13 cycles were potentially missing and 18 (5+13) cycles are visible (Table 1). Starting with the minimum number (11 cycles), we created a new growth series with 2 kg body mass at age 0, 6129 kg at age 11 ( = first preserved growth mark), 7980 kg at age 12 ( = second preserved growth mark), and so on (Table 2, Table S1). Using this new growth data, we conducted a non-linear regression analysis to find out whether it fits to the vBGM, LGM, or CRGM. We then manually increased the estimated number of missing cycles by one, created new growth series, and carried out curve fittings until the maximum number of missing growth cycles was reached (for *Apatosaurus* sp. femur BYU 601-17328, 11, 12, and 13 missing cycles were tested). All regression models obtained from manually testing different constant numbers of missing cycles for a specific growth model were ranked according to their AIC values. An AIC (Akaike Information Criterion) value is a measure of the *relative* goodness of fit of raw data to a statistical model [74]. The statistically best from the manually tested growth models was selected according to the method given in Burnham and Anderson [75] and with respect to biological plausibility (for details see section on model selection, below).

**Table 2:** Results of growth curve fitting for Sauropodomorpha.

Taxon	Spec. no.	Visible cycles	Model and approach	M_0_(kg)	A (kg)	A_est_ (kg)	g_est_ (kg/year)	Missing cycles	AD (years)	AA (years)	ASM (years)	MGR (g/day)	Residual s.e.	df	AIC	df
*Plateosaurus engelhardti*	IFG 192.1	12	LGM 1	2	1,587		0.767***	4.4	16.4	16.4	10.4	819.2	52.9	11	133.9	2
		12	LGM 2	2	1,587		0.642***	7	19	19	13.1	680.5	40.2	11	137.5	2
indet. mamenchisaurid	SGP 2006/9	22	LGM 2	2	25,075		0.233***	9	31	45	20.2	3907.7	750.9	21	374.6	2
*Apatosaurus* sp.	SMA 0014	20	LGM 2	2	20,206		0.285***	8	28	34	21.1	4024.1	701.5	19	339.6	2
*Apatosaurus* sp.	BYU 601–17328	18	LGM 2	2	18,178		0.364***	13	31	31	19.0	4421.4	323.5	17	278.2	2
indet. diplodocid	MfN.R.2625	9	LGM 2	2		4,753***	0.327***	15	24	33	22.9	901.1	56.78	7	113.6	3
indet. diplodocid	MfN.R.NW4	16	LGM 2	2		18,463**	0.203***	7	23	43	24.7	2335.1	612.5	14	271.6	3
*Camarasaurus* sp.	CM 36664	9	LGM 2	2	14,247		0.467***	17	26	27	20.7	4188.8	380.8	8	151.7	2

To reduce the number of estimated parameters and enable convergence of the fitting procedure to the data when the number of preserved growth marks is small in comparison to the number of parameters, we always considered different versions of regression models for the vBGM, LGM, and CRGM. The most general model version estimated all parameters of equations (2), (3), or (4). The next two versions assumed either a fixed *A* (the body mass estimated for the individual, Table 1) or a fixed *M_0_* (the mass at the first visible cycle for FT1 or a hatchling mass of 2 kg for FT2). The last version considered fixed *M_0_* and *A*, and it estimated only the remaining one (*g*, for vBGM and LGM) or two parameters (*g*, *m*, for CRGM) of the growth model under study. In total, we fitted four versions of each of the three growth models to the histological growth series of each of the seven specimens, resulting in a total of 12 growth functions considered for each of the specimens. Each of these 12 growth functions was tested for each specimen using the two curve-fitting techniques.

All regression analyses were carried out with the free software R statistics (version 2.9.0, http://www.R-project.org). We applied the function “nls” from the *nls* package (non linear regression analysis) for model fitting.

### Model selection

The two fitting approaches and three growth models with four model versions considered for each specimen revealed several regression functions describing the growth in body mass in the individuals. We therefore utilized the AIC approach as implemented in the *nls* package to find the best of these regression models for each of the specimens. All regression models found for a specimen were ranked according to their AIC values, and the best model with the lowest AIC (min(AIC)) was identified. We calculated ΔAIC for each model (AIC- min(AIC)) and followed the evaluation approach suggested by Burnham and Anderson [75]. ΔAIC scores less than 2 suggest well-supported models, scores between 2 and 10 suggest moderate support, a score >10 suggests a weakly supported model relative to the alternative model (with the lowest AIC).

Models that were biologically unrealistic were excluded *a priori* from the model selection process. Biological criteria used included the estimated asymptotic mass (*A*) that was unrealistic for the taxon, the estimates of the number of missing cycles that clearly ranged outside the minimum and maximum interval calculated for the specimen (only applicable to models derived from FT1, where the number of missing cycles is read off from the curve at hatchling weight, Table 2), and estimated hatchling weights that were larger than 10 kg or smaller than 2 kg (only applicable to models from FT2 and if *M_0_* was not fixed in the model version).

### Treatment of growth marks in the EFS

**Figure 2:**
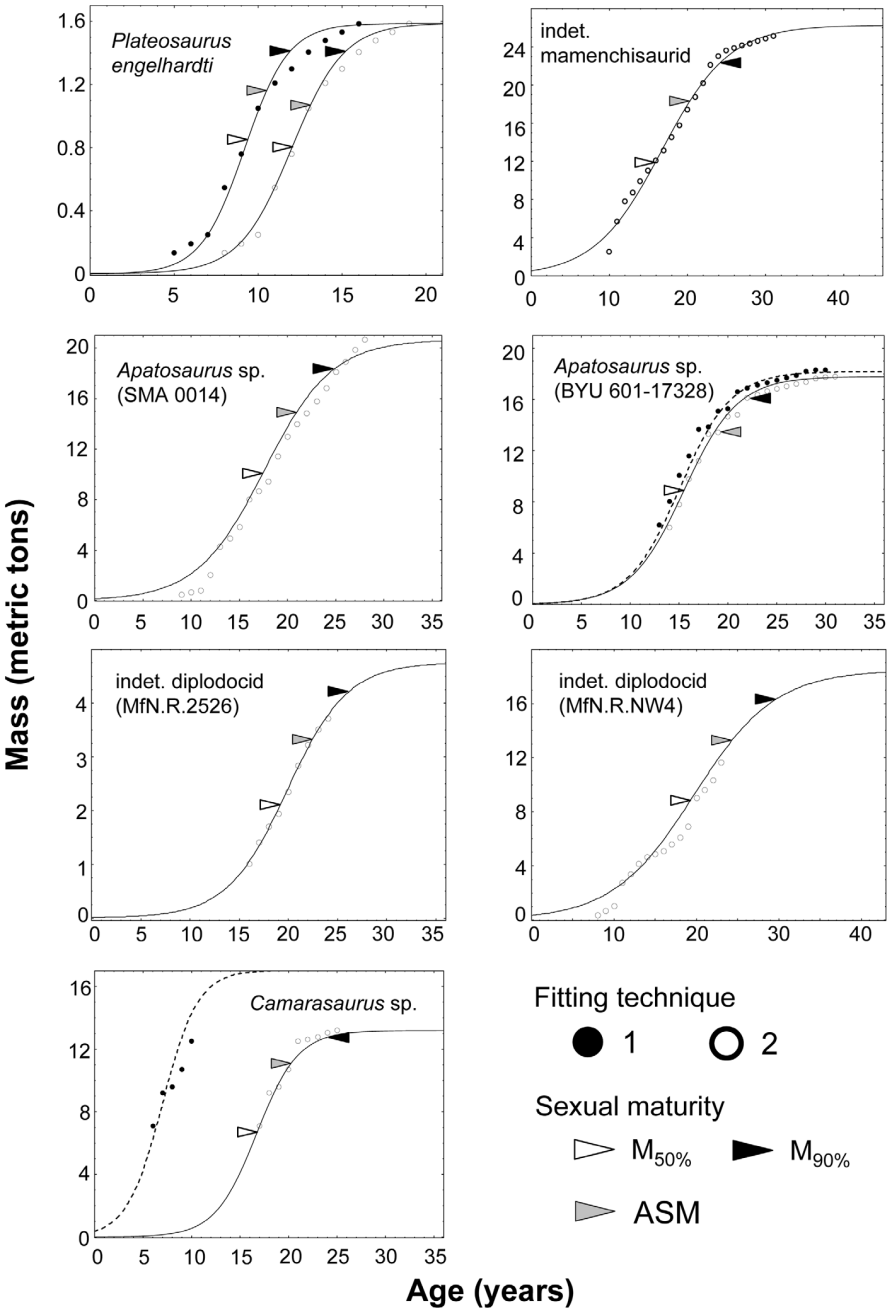
Logistic growth models established for Sauropodomorpha individuals. Two curve fitting techniques were applied to construct growth curves from the growth mark record laid down in long bones (Table 1). Only the statistically best supported models are shown, except for *Apatosaurus* sp. (BYU 601-17328) and *Camarasaurus* sp. For these two individuals, the logistic growth models (dotted line) based on the non-EFS growth marks only are also shown. Triangles refer to different estimates of age at sexual maturity. Age at the inflection point of the growth curve (white triangle) is the lower limit (50% of asymptotic mass = M_50%_), whereas age at 90% asymptotic mass (black triangle) is the upper limit (M_90%_) of the onset of reproduction. The grey triangle (*ASM*) indicates the arithmetic mean of these time spans and corresponds to the arithmetic mean of the earliest and latest age at which first reproduction could have occurred. Parameter values of the best models are summarized in Table 2. Model selection was based on the AIC approach [75].

Because it has been argued that growth marks in an EFS should not be counted as annual marks [76], we additionally modelled growth in the two fully-grown individuals *Apatosaurus* sp. BYU 601-17328 and *Camarasaurus* sp. as described before but we excluded all growth marks in the EFS from the analysis. For both individuals, the non-EFS growth record consisted of only five growth marks which is relatively few compared to the number of growth marks in the EFS (Table 1). Curve fitting was only successful for *Apatosaurus* sp. For the latter specimen the best statistical model was the LGM that was found by FT2 (Figure 2). It suggested an asymptotic mass *A* = 18,524 kg (p<0.01), a growth rate *g* = 0.382 kg/year (p<0.01) and 13 missing growth cycles. The inflection point predicted by this model was 15.080 years. For the *Camarasaurus* sp. individual, FT1 resulted in a non-significant model that assumed an asymptotic mass *A* = 17,032 kg (p = 0.07), a growth rate *g* = 0.547 kg/year (p = 0.07) and five missing growth cycles. The estimated high asymptotic mass of *Camarasaurus* contradicts bone histology. The individual shows an EFS and thus indicates that the individual is fully-grown, whereas the LGM model predicts a considerable increase in body mass after its death (Figure 2). Because the model found for *Apatosaurus* sp. was biologically reliable and it differed only slightly from the statistically best model found by including the growth marks in the EFS (Table 2, Figure 2), we assume in this study that potential errors in the analysis of life history traits introduced by including growth marks in the EFS are small.

### Establishing allometries for Sauropodomorpha

From the best growth curves obtained for each of the seven specimens, we established allometries between body mass and age at death, asymptotic age, age at sexual maturity, and maximum growth rate by applying ordinary linear least squares regression analysis on log-log-transformed data. If the best model was found by FT1, age at death of the individual was estimated as the number of visible growth cycles (Table 1) plus the estimated number of missing cycles. For FT2, the individual age at death was directly read off from the growth curve (Table 2). The individual ages at death obtained from the long bones used in this study, however, most probably overestimate the life expectancy in populations or even species. All individuals studied had survived the juvenile stage before they died (HOS >7, Table 1). Mortality during the juvenile stage was most likely significantly higher than mortality during the adult stage in populations of large Sauropodomorpha, because size increases with increasing age of individuals, and smaller individuals experience higher mortalities from predators than larger individuals [12].

For asymptotic age of individuals, the time when the individual has reached 99% of its asymptotic mass, *A* was read off from the best growth curve, and for FT1, the estimated number of missing growth cycles was added to this time.

To estimate the age at sexual maturity for each of the studied individuals, we calculated *ASM* as the arithmetic mean of the age at which the inflection point of the best growth curve is found and the age at which 90% of the asymptotic mass *A* was observed under this growth model.

To estimate maximum growth rates of the individuals [77], we used the age from the inflection point of the growth curve (*t_i_*), calculated both the largest integer age smaller than *t_i_* (*t_lower_*), and the smallest integer age larger than *t_i_* (*t_upper_*), and determined the increase in body mass within the interval [*t_lower_*, *t_upper_*] from the growth curve as estimate of the annual maximum growth rate. For a comparison of maximum growth rates of Sauropodomorpha with the allometries of Case [77] for extant vertebrates, we converted these annual maximum growth rate estimates (in kg) for Sauropodomorpha to grams per day assuming a constant daily rate over the year. An analogous approach to estimate daily maximum growth rates [77] is used when it is calculated from the largest increase in body mass recorded between two growth cycles.

To roughly assess potential errors in estimates of maximum growth rates, we adopted the approach of Case [77] that he developed to facilitate a comparison of his maximum growth rates with the rates given in Ricklefs [49], [52]. For each of the sauropodomorph individuals, we calculated from the best growth curve the average growth rate within the time span confined by 10% and 90% of asymptotic mass of the individual (length of the quasi-linear phase of growth in birds). Mass increments within this time span were averaged on a yearly base.

## Results

### Growth models

While the vBGM and CRGM were only applicable to a few specimens, the LGM was applicable to all Sauropodomorpha individuals studied. The vBGM was only successfully fitted to *Plateosaurus*, *Apatosaurus* BYU 601-17328, and the indeterminate mamenchisaurid, and the numbers of model parameters estimated differed between these individuals. For *Plateosaurus* and the indeterminate mamenchisaurid the best fits in terms of AIC were obtained for the model version that only estimates parameter *g*, whereas for *Apatosaurus* BYU 601-17328, the best model version estimated *g* and *A* (equation 2). The CRGM was only successfully fitted to *Apatosaurus* BYU 601-17328 and *Camarasaurus*. The specific CRGM model versions estimated parameters *m*, *g* and *A* (equation 4). The number of missing cycles derived from the CRGMs for *Apatosaurus* BYU 601-17328 and *Camarasaurus* was between 2 and 3. This small number strongly contradicts the retrocalculation from histological data (11–13 for *Apatosaurus* BYU 601-17328, 16–26 for *Camarasaurus* sp., see Table 1) and questions the biological plausibility of the CRGMs for both individuals.

In contrast, the LGM was successfully fitted to all seven Sauropodomorpha. For *Plateosaurus* and *Apatosaurus* BYU 601-17328, the LGM was clearly preferred over the respective vBGM (ΔAIC >10) in terms of AIC, whereas for the indeterminate mamenchisaurid the LGM was only moderately better supported than the vBGM (2< ΔAIC <10). The best fits of growth records to the LGM were obtained for the model version that only estimated parameter *g*, except for the two indeterminate diplodocids MfN.R.NW4 and MfN.R.2625, where parameters *g* and *A* were estimated (equation 3). For all sauropodomorph individuals, except for *Plateosaurus* (2< ΔAIC <10), the LGM found by FT2 was much stronger statistically supported in terms of AIC than the model derived from FT1 (ΔAIC >10).

The best growth curve estimates for all Sauropodomorpha individuals studied here are thus LGMs. They are shown in Figure 2 and Table. Life history traits derived for the individuals from growth curves are given in Table 2.

### Allometry of age at death versus body mass

#### Model results

**Figure 3:**
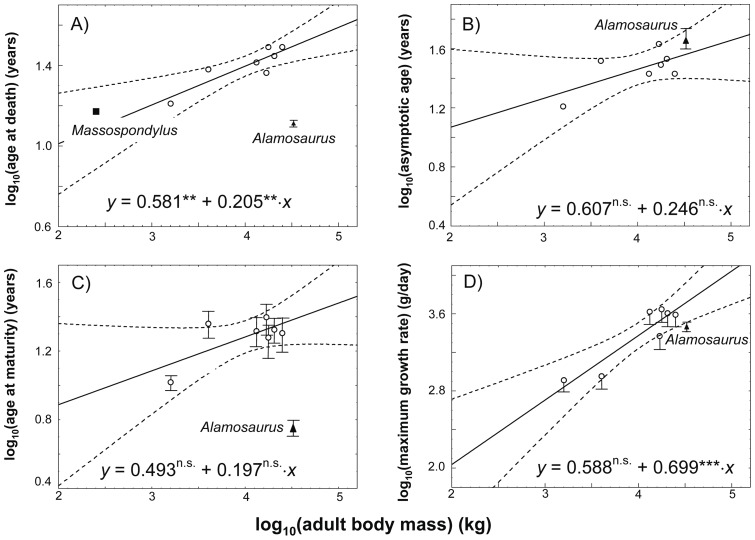
Allometries derived for Sauropodomorpha and comparison with the literature data. **A)** Age at death vs. body mass, **B)** asymptotic age vs. body mass, **C)** age at sexual maturity (*ASM*) vs. body, mass and **D)** maximum growth rate vs. body mass. Regression lines (continuous line) and 95% confidence interval (dashed lines) are shown. Filled square in **A)**: age at death of *Massospondylus* [21]. Filled triangles with error bars in all graphs: estimates of life history traits for *Alamosaurus* from Lehman and Woodward [34]. Error bars in **C)**: lower limit (age at inflection point of the growth curve), and upper limit (age at which 90% of the asymptotic mass is reached, [49], [52], see Figure 2C) of age at sexual maturity. Error bars in **D)**: average daily increase in body mass during the quasi-linear phase (between 10% and 90% of the asymptotic mass, [77]). Statistics of allometric functions: A) R^2 = ^0.841, intercept p = 0.010, slope p = 0.003; B) R^2^ = 0.538, intercept p = 0.271, slope p = 0.061; C) R^2^ = 0.494, intercept p = 0.230, slope p = 0.079; D) R^2^  = 0.895, intercept p = 0.230, slope p = 0.001. ^n.s.^ p>0.05, * p≤0.05, ** p<0.01, *** p<0.001.

The age at death of Sauropodomorpha studied significantly increases with increasing body mass (log-log-plot, Figure 3A; intercept p = 0.010, slope p = 0.003). The coefficient of determination of the linear regression function is high (R^2^ = 0.841), reflecting a low variability in residuals. Even the estimate of age at death obtained for *Plateosaurus* from the second best model (FT2, Table 2) falls within the 95% confidence interval of the regression line, but the estimated age at death of *Camarasaurus* was slightly lower than the lower limit of this confidence interval (Figure 3A).

#### Comparison with histological results

Estimates of age at death obtained from growth models coincided well with estimates derived from histology. This was expected when applying FT2 because this approach makes use of the *a priori* estimated minimum and maximum number of missing cycles to find the best fitting growth model. Only FT1 estimates the number of missing cycles without any previous information, but this approach was only applicable to *Plateosaurus*. For *Plateosaurus*, 4.4 missing cycles are predicted by the LGM, resulting in an age at death of the individual of 16 years, which coincides well with an age at death between 12 and 21 years that is suggested by bone histology (Table 3).

**Table 3:** Comparison of growth models with histological data for Sauropodomorpha.

Taxon	Spec. no.	AD (years)		AA (years)		ASM (years)			
		Histology	Growth model	Histology	Growth model	Histology	Growth model		
							M_50%_	M_90%_	*ASM*
*Plateosaurus engelhardti*	IFG 192.1	15–21	16.4–19	no EFS	16.4–19	9–17	9.4–11.1	11.4–15.1	10.4–13.1
indet. mamenchisaurid	SGP 2006/9	26–31	31	**26**–**31**	**45**	**≤9**	**15.7**	**24.7**	**20.2**
*Apatosaurus* sp.	SMA 0014	22–28	28	no EFS	34	**≤8**	**17.6**	**24.6**	**21.1**
*Apatosaurus* sp.	BYU 601–17328	29–31	31	29–31	31	≤13	15.5	22.5	19.0
indet. diplodocid	MfN.R.2625	17–24	24	no EFS	33	≤15	18.9	26.9	22.9
indet. diplodocid	MfN.R.NW4	18–23	23	no EFS	43	13–18	19.7	29.7	24.9
*Camarasaurus* sp.	CM 36664	20–28	26	16–26	27	≤17	16.7	24.7	20.7

### Allometry of asymptotic age versus body mass

#### Model results

The asymptotic age of Sauropodomorpha non-significantly increases with body mass (log-log-plot, Figure 3B; intercept p = 0.271, slope p = 0.061). The coefficient of determination of the linear regression function is only moderate (R^2 = ^0.538), reflecting a high variability in residuals. In accordance with the low quality of the fit, the estimated asymptotic ages of all Sauropodomorpha (including the estimate from the second best models for *Plateosaurus*, Table 2) range within a broad 95% confidence interval.

#### Comparison with histological results

Asymptotic ages obtained from growth models for Sauropodomorpha are only comparable with histological estimates when the individual is verifiably fully-grown, as indicated by the presence of an EFS. For the *Apatosaurus* BYU 601-17328 individual and the *Camarasaurus* individual the models predicted an asymptotic age which is more or less consistent with histology (Table 3). The sum of the minimum (maximum) number of missing cycles, the number of visible growth marks, and the number of EFS cycles suggests that *Apatosaurus* individual BYU 601-17328 died at an age of 29 (31) years. This coincides well with the age of 31 year predicted by the growth curve; 31 years is the age at which 99% of the asymptotic mass is reached. Analogous calculations suggest that the *Camarasaurus* individual died at the age of 16 up to 26 years, which is still close to the 27 years predicted by the growth model. In contrast, the indeterminate mamenchisaurid individual had an estimated age at death of 31 years based on histology and shows an EFS, but the asymptotic age predicted from the growth curve is 45 years and thus is most probably inconsistent with histology. Because only two of the three fully-grown individuals studied had asymptotic ages predicted from growth curves, that are more or less consistent with those suggested by bone histology, we conclude that additional individuals are needed to statistically support the accuracy of the regression function on asymptotic age in Sauropodomorpha.

### Allometry of age at sexual maturity versus body mass

#### Model results

Age at sexual maturity of the Sauropodomorpha as defined in this study (*ASM*) non-significantly increases with increasing body mass (log-log-plot, Figure 3C; intercept p = 0.230, slope p = 0.078). The coefficient of determination of the linear regression function established was the lowest (R^2^ = 0.494) of all allometries. Estimates obtained for each of the individuals are located within the broad 95% confidence interval, except for the indeterminate diplodocid MfN.R.NW4 individual, which is older at sexual maturity than the upper limit of the 95% confidence interval.

#### Comparison with histological results

A direct comparison of ages at sexual maturity with those derived from the histological examination of the bones was only possible for two specimens (*Plateosaurus*, diplodocid individual MfN.R.NW4, Figure 2, Table 3). In the *Plateosaurus* specimen, the onset of type E bone tissue coincides with the sixth to the eighth growth cycle, whereas the inflection point from both growth models is located between the fifth and sixth mark, the upper limit of the quasi-linear phase of growth is reached between the eighth and ninth mark, and *ASM* is in-between these limits. For the diplodocid individual MfN.R.NW4, the eleventh growth mark is located in the early type E bone tissue, and the inflection point of the growth curve closely coincides with this location (between the 12^th^ and 13^th^ mark). In contrast, both *ASM* and the age at which 90% of the asymptotic mass is reached by this individual are clearly outside the growth record.

For both *Apatosaurus* individuals (BYU 601-17328 and SMA 0014), *Camarasaurus*, the indeterminate diplodocid MfN.R.2625 and the indeterminate mamenchisaurid, no histological record of sexual maturity occurred in the bone tissue because the transition from type D to type E bone tissue had been destroyed by medullary expansion or secondary remodeling. In these cases, we would expect that the number of missing cycles estimated by the growth model should be higher than *ASM* predicted by the growth model. In contrast to our expectation, the growth models predicted that *ASM* is documented in the growth record for these five Sauropodomorpha, and only when taking the inflection point of the growth model as age at sexual maturity, the congruence between histology and growth models was slightly better for three individuals out of five. When using the inflection point as indicator of sexual maturity, sexual maturity was before the growth record started for *Apatosaurus* BYU 601-17328, *Camarasaurus*, and the indeterminate diplodocid MfN.R.2625, but for *Apatosaurus* SMA 0014 and the indeterminate mamenchisaurid sexual maturity was still predicted at an age covered in the growth record (Figure 2, Table 3). However, since the inflection point is not documented in the growth record of these five individuals, this critical information also was unavailable for the non-linear regression analysis, thus hampering the fitting process. Because only one out of seven Sauropodomorpha revealed estimates of *ASM* from our allometry consistent with bone histology, we conclude that additional specimens are needed to statistically support the accuracy of the regression function on age at sexual maturity.

### Allometry of maximum growth rate versus body mass

#### Model results

Maximum growth rate was estimated for the year of the inflection point of the growth curve. Maximum growth rate increased significantly with body mass (log-log-plot, Figure 3D; intercept p = 0.230, slope p = 0.001), but the intercept of the regression line was not significant. The coefficient of determination of the linear regression function was the highest of all allometries (R^2^ = 0.895). Estimates obtained for each of the individuals under the best growth model fall within the 95% confidence interval, and even the estimate derived for *Plateosaurus engelhardti* (IFG 192.1) from the second-best model falls within this interval. When using the average growth rate during the quasi-linear phase of growth as a further estimate of maximum growth rate, five out of the seven Sauropodomorpha still fall within the 95% confidence interval of the regression line. Only the maximum growth rates of *Camarasaurus* and of the indeterminate diplodocid MfN.R.2625 were somewhat lower than the lower limit of this interval.

Both the maximum growth rates of Sauropodomorpha estimated from the inflection point and from the quasi-linear phase of the growth models were in the realm of precocial birds ([77], Table 2, Figure 3). The regression line of precocial birds of Case [77] is placed within the 95% confidence interval of Sauropodomorpha. His eutherian mammal regression line is clearly above this 95% confidence interval, and his reptile line is below it. The latter observation still applies even if the lower maximum growth rates derived from the quasi-linear phase of growth are used.

#### Comparison with histological results

Sander et al. [17] made a preliminary attempt to estimate the maximum growth rate of *Apatosaurus* SMA 0014 based on the largest observed increase in body mass between two growth marks. This estimate is 2,500 kg/year (6,849 g/day), which is not fundamentally higher than the “inflection point” estimates for the two *Apatosaurus* individuals (SMA 0014: 4,024.11 g/day; BYU 601-17328: 4,421.37 g/day) and “quasi-linear phase” estimates (SMA 0014: 2,939.54 g/day; BYU 601-17328: 3,248.04 g/day). However, all estimates are an order of magnitude higher than under the reptile model of Case [77] (SMA 0014: 346.67 g/day; BYU 601-17328: 313.69 g/day).

The growth records of both *Apatosaurus* individuals studied herein are poor and suffer from different problems that hamper the fitting of growth curves. While the record of SMA 0014 includes only an indistinct inflection point, none is documented in BYU 601-17328. This lack of important information about the transition from growth acceleration to growth deceleration in *Apatosaurus* may explain the differences in the maximum growth rate observed in the growth record and predicted by the growth models.

## Discussion

### Test of cyclicity of growth mark records

In this study, we successfully applied various mathematical growth models to seven individuals pertaining to five different sauropodomorph taxa. The first aspect we would like to discuss is the implication of model fitting to the question of the annual nature of histological growth marks in dinosaurs. While this is generally accepted [17], [20], [26] and additional support has come from a recent study on extant mammals [27], there have been dissenting voices [78]. Growth models can be used to indirectly test the annual nature of growth marks. Growth models are designed to describe, among other parameters, the increase in body mass over time, and raw data underlying growth models are time series of body masses. In the case of growth marks in dinosaurs, we want to test whether these growth marks were deposited at regular vs. random intervals. The growth model has a good fit to a data set of growth marks vs. body mass, suggesting that the growth marks are indeed cyclical. The only biologically plausible interval for growth marks is the year, at least in very large dinosaurs such as the Sauropodomorpha. In other words, if growth marks were to reflect random decreases of growth during the life of the animal, standard growth models that describe a systematic increase in body mass after birth in extant animals would not be applicable to data sets derived from dinosaurs.

### Growth model selection and curve fitting techniques

At the outset of this study, we had assumed that growth models making no *a priori* assumption about the position of the inflection point of the growth curve (CRGM) would be more suitable for estimating life history traits and in particular ages at which growth starts to decelerate (inflection point of the growth curve) of extinct species than models with a predetermined inflection point (vBGM, LGM, and GGM). However, the LGM turned out to be the best model for describing the growth in body mass in Sauropodomorpha. The CRGM was only applicable to the *Apatosaurus* BYU 601-17328 and the *Camarasaurus* individuals, but models performed rather poorly because the estimated number of missing cycles strongly contradicted the histological data (Table 1). Because one more parameter has to be estimated during the fitting process (parameter *m*) for the CRGM in comparison to the LGM and the vBGM, the failure of fitting the CRGM is possibly caused by an insufficient number of growth marks in comparison to the total number of model parameters estimated. If so, we would expect that fitting a CRGM would be successful for individuals with a growth record covering a broad range of body masses in the ontogeny of the individual and showing many growth cycles, e.g., for the indeterminate mamenchisaurid (22 visible cycles) and *Apatosaurus* SMA 0014 (20 visible cycles). For these two individuals, however, the LGM was the best. The CRGM was only successfully fitted to two individuals showing a poor growth record regarding the number of growth marks preserved and the body mass range covered (*Camarasaurus*, 9 visible cycles, HOS 12, *Apatosaurus* BYU 601-17328, 5 non-EFS visible cycles, HOS 12). This discrepancy suggests that technical reasons are not responsible for the poor performance of the CRGM for Sauropodomorpha and favors the applicability of standard growth models (vBGM and LGM) for this taxon.

The LGM turned out to be the best growth model for all Sauropodomorpha studied. This model was also successfully applied to multi-individual growth data by Erickson et al. [32] of *Massospondylus carinatus* (nine femora) and *Apatosaurus exelsus* (four scapulae). In the study of Lehman and Woodward [34], the vBGM was successfully applied to one multi-individual growth data set, that of *Apatosaurus exelsus* (re-analysis of the scapulae used by Erickson et al. [32]) and three single-individual data sets, i.e., *Alamosaurus sanjuanensis* (based on a single humerus), *Janenschia robustus* (based on a single femur), and an indeterminate sauropod from England (based on a single pubis). Note that we are discussing the model fit to the data in these studies, not the reliability of the results (see introduction).

However, contrary to our study, neither Erickson et al. [32] nor Lehman and Woodward [34] had concurrently tested the LGM and the vBGM for any time series of body masses and were thus unable to objectively rank the two growth models based on their AIC. While Erickson et al. [32] do not provide any argument for using the LGM, Lehman and Woodward [34] give two heuristic arguments for their preference of the vBGM over the LGM for *Apatosaurus exelsus*: First, the vBGM performs well for the closest living relatives of dinosaurs (birds, [49]) as well as for the largest living terrestrial animals, the elephants ([40], [79], but see [45], [70]). The second argument of Lehman and Woodward [34] for using the vBGM was that it produces estimates for life history traits that were consistent with predictions derived from allometries that had previously been obtained from other dinosaurs. We believe that the second argument of these authors amounts to circular reasoning.

The LGMs established for individuals by curve-fitting technique 2 (FT2) always performed better than the approach that explicitly estimated the number of missing cycles (FT1), except for *Plateosaurus*. For this individual, FT1 produced a statistically better supported model than FT2. The LGM derived from FT1 was only slightly better in terms of AIC (ΔAIC = 3.6) than the model derived from FT2, and both models were very well supported by bone histology. The growth record of *Plateosaurus* is exceptionally good for a sauropodomorph individual because it covers a very broad ontogenetic range of body masses, and only a few growth cycles are lost to resorption in the inner part of the bone.

Our results suggest that the application of FT2 may eventually rely on the availability of precise estimates of the number of cycles missing in the inner part of the bone. This could eventually limit the applicability of this curve-fitting technique to other Sauropodomorpha (but not to other dinosaurs). However, we observed that when manually increasing the number of potentially missing cycles stepwise (FT2), we either found a clear minimum AIC or a hyperbolic decrease in AIC values. Thus, a stepwise increase in the number of missing cycles (starting with the minimum estimated number of cycles) until a minimum AIC is reached could at least be used to create models that are testable with respect to histological and biological plausibility when estimates of the number of cycles missing in the inner part of the bone are difficult to obtain.

For all sauropodomorph specimens studied, except for *Plateosaurus engelhardti*, growth models were not consistent with bone histology (HOS 8 as indicator of sexual maturity) when using *ASM* to estimate age at sexual maturity. In contrast, the inflection point of the growth model coincided with HOS 8 for the diplodocid individuals MfN.R.NW4 and MfN.R.2625, *Apatosaurus* BYU 601-17328, and *Camarasaurus*, and this could support the inflection point concept for sexual maturity. However, many more specimens need to be studied to clarify whether *ASM* or the inflection point is the better estimator of the age at sexual maturity in Sauropodomorpha.

### Consistency of allometries with published life history traits of other Sauropodomorpha

To compare the allometries of life history traits established in this study with those for other Sauropodomorpha, only two reliable studies are available (see the introduction). Because allometries can strongly differ between extant taxa [4], [77], [80], we only discuss the applicability of allometries to Sauropodomorpha but not to dinosaurs in general. In addition to the sauropod *Alamosaurus* taken from Lehman and Woodward [34], we selected the basal sauropodomorph *Massospondylus* from Chinsamy-Turan [21] for comparison. This taxon has a very good multi-individual growth record as already noted by Chinsamy [23] and Erickson et al. [32].

Three vBGMs had been established by Lehman and Woodward [34] for *Alamosaurus* from which the authors derived age at death, asymptotic age (*A*), age at sexual maturity, and maximum growth rate of the individual. The asymptotic age predicted by these models for the *Alamosaurus* dataset fits well within the 95% confidence interval of our regression (Figure 3B). The maximum growth rate of this individual is slightly lower than the lower limit of this interval (Figure 3D), whereas its age at death (Figure 3A) and age at sexual maturity (i.e. at the inflection point) is much too low (Figure 3C) when compared to our results. While all individuals studied here have passed sexual maturity (>HOS 8) when they died, the *Alamosaurus* individual is a subadult [34], of HOS 7. Thus age at death may have been lower than predicted because our regression is based only on ontogenetically older individuals (≥ HOS 10) than the *Alamosaurus* individual.

The age at sexual maturity that Lehman and Woodward [34] derived from the inflection point of their growth models for the *Alamosaurus* individual was much lower than expected from our allometry. This difference can be largely attributed to the different growth models applied. Lehman and Woodward [34] fitted a vBGM and thus indirectly assumed that the inflection point of their fitted growth curve is reached earlier than under our LGM (at 30 vs. 50% of the asymptotic body mass). We therefore re-evaluated the histological growth record given in Lehman and Woodward [34] and found that the inflection point is located one mark before the last documented growth mark. There are nine growth marks preserved, four to five marks are missing, giving an age of 12–13 years for the revised inflection point vs. five to six years predicted by the vBGM in the original study. The revised estimates of age at the inflection point and maximum growth rate (1,403 kg/year or 3,843 g/day vs. the original 1,090 kg/year or 2,986 g/day) of *Alamosaurus* are much closer to the ages at sexual maturity predicted by our allometries from growth curves, and now fit into the 95% confidence interval of the regression lines.

For *Massospondylus*, Chinsamy-Turan [21] estimated an age of 15 years at death. This age coincides very well with our allometry, although the estimated adult body mass of this basal sauropodomorph is an order of magnitude smaller than that of *Plateosaurus*.

We conclude that other studies on Sauropodomorpha support the allometries established in this study and that these can at least be used to approximate life history traits of other individuals of this taxon. An exception is the basal sauropodomorph *Plateosaurus* for which predictions are less reliable because of its strong developmental plasticity [24].

### Aging, maturation, and growth in Sauropodomorpha

**Figure 4:**
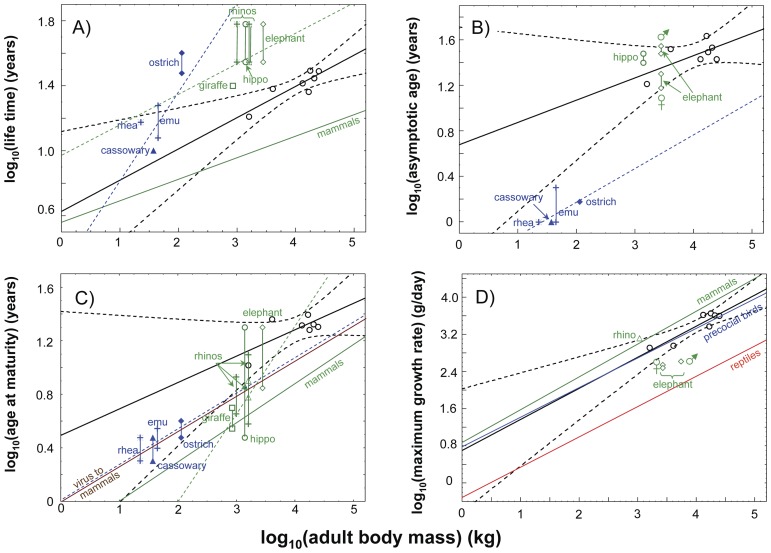
Comparison of life history traits of Sauropodomorpha, extant ratites and extant mammalian megaherbivores. All regression lines for extant species are extrapolated to the body masses of Sauropodomorpha. In all panels the dashed blue lines and dashed green lines are regression lines for ratites and megaherbivores, respectively. These regression lines were calculated based on all data points for the respective taxon. None of the dashed regression lines is statistically significant. Open circles in **A)** to **D)** are Sauropodomorpha. Data for megaherbivores are from Owen-Smith [50] and those for ratites are from del Hoyo [81]. Symbol explanation for megaherbivores (green): elephant: *Loxondonta africana* (open diamonds), hippo: *Hippopotamus amphibius* (open circles), giraffe: *Giraffa camelopardalis* (open squares), rhinos: *Rhinoceros unicornis* (crosses), *Diceros bicornis* (asterisks), *Ceratotherium simum* (open triangles). Symbol explanation for ratites (blue): ostrich: Struthio camelus (filled diamonds), emu: *Dromaius novaehollandiae* (crosses), cassowary: *Casuarius casuarius* (filled triangles), rhea: *Rhea americana* (asterisks). **A)** Life expectancy in wild populations. Vertical bars connect minimum and maximum life expectancies for elephant, hippo, rhinos, and emu. Ostrich data are from captive animals; green line: allometry of life expectancy in mammals [4]. **B)** Asymptotic age in wild populations. Bars: minimum and maximum values for elephant (male, female), hippo and emu. **C)** Age at sexual maturity in wild populations. Vertical bars connect minimum and maximum values for all species; brown line: virus-to-mammals allometry of maturation time [4]; green line: allometry of age at first conception in mammals [50]. Age at sexual maturity of Sauropodomorpha is based on *ASM* of the best growth model (Figure 2C, grey triangle). **D)** Maximum growth rate. Sauropodomorpha (black line, 95% confidence interval dotted), eutherian mammals (green line, [77]), precocial birds (blue line, [77]), reptiles (red line, [77]). Growth rates of Sauropodomorpha are in the realm of precocial birds. The regression line of eutherian mammals is clearly above the 95% confidence interval for Sauropodomorpha, and the line for reptiles is well below the confidence interval. Maximum growth rates of Sauropodomorpha are based on the inflection points of the best growth models (Figure 2D).

At the outset, we would like to remind the reader that because of uncertainties about the original thickness of the cortex, maximum growth rate is overestimated and the values of all ages are underestimated. Growth curves and allometries in this study suggest that the life history of Sauropodomorpha is similar in several aspects to the life history of extant ratites, the largest herbivorous terrestrial birds, and of mammalian megaherbivores (Figure 4). As seen in many reptiles and large mammals, such as female elephants [40], [51], Sauropodomorpha reached sexual maturity well before they were fully-grown (as already suggested by Sander [38] and corroborated by the histological ontogenetic stages from Klein and Sander [16]). While ages at sexual maturity of ratites and megaherbivores are consistent with the virus-to-mammals allometry of Blueweiss [4] and are larger than predicted by an allometry on mammals only [79], Sauropodomorpha were older at sexual maturity than scaled-up ratites and than average mammals (Figure 4C). Megaherbivores, however, mature later than average mammals [50]. Thus, small Sauropodomorpha (e.g. *Plateosaurus engelhardti*) showed an age at sexual maturity as seen in the largest similar-sized megaherbivores, and the larger Sauropodomorpha (all other Sauropodomorpha studied) possibly matured earlier than scaled-up megaherbivores. Because the data set for extant megaherbivores is small (the regression line in Figure 4C is not significant), the latter observation is not statistically supported.

Asymptotic ages of Sauropodomorpha resemble the ages seen in extant mammalian megaherbivores, but were higher than those of scaled-up ratites (Figure 4B). The maximum growth rates of Sauropodomorpha fit well to the average found in scaled-up precocial birds, but are lower than the rates of average mammals. Maximum growth rates of megaherbivores (Figure 4D), however, either fit within the 95% confidence interval of Sauropodomorpha, like in the black rhino (*Diceros bicornis*), or were considerably lower than the lower limit of this interval, like in the African elephant (*Loxodonta africana*), indicating that on average ratites (scaled-up), megaherbivores (scaled-up) and Sauropodomorpha have similar maximum growth rates.

Ages at death derived for the adult Sauropodomorpha individuals in this study and life expectancies found in populations of ratites are above the allometry of mammals ([4], Figure 4A). An exception is the ostrich, *Struthio camelus*, which shows a higher life expectancy (Figure 4A), but those data originate from captive animals. Mammalian megaherbivores have higher life expectancies than ratites (scaled-up ratites could have higher life time expectancies than megaherbivores as suggested by the non-significant regression line in Figure 4A) and the ages of death observed in sauropodomorphs (Figure 4A). Keeping in mind that ages at death of the adult Sauropodomorpha individuals studied most probably overestimate the real average in populations, the life expectancies of Sauropodomorpha populations should have been even lower than in scaled-up ratites and scaled-up megaherbivores.

To sum up, aging, maturation, and growth patterns in Sauropodomorpha show a combination of strategies seen in recent megaherbivores and today's largest birds. This suggests that similar environmental factors have shaped the life history of extinct Sauropodomorpha and of extant large animals. The higher ages at sexual maturity seen in larger Sauropodomorpha in comparison to today's megaherbivores and ratites could have been compensated for by a higher annual number of offspring facilitated by a larger body size [12], [13]. Viviparity could have favored the distinct K-strategy found in extant mammalian megaherbivores that is absent in extant oviparous ratites and most probably absent in extinct oviparous Sauropodomorpha [10], [12], [13].

In conclusion, we provided the first comprehensive analysis of histologically derived single-individual growth data sets of body mass vs. age for sauropodomorph dinosaurs using different growth models and fitting techniques. Based on the best-fitting models, we derived allometries between body mass and life history traits for Sauropodomorpha. While the data base for our growth curves and allometries are admittedly limited, great potential is apparent in the data and their interpretation. For the first time, we have more than anecdotal evidence for the commonly asked question of the age at death of Sauropodomorpha, for their ages at sexual maturity, and for their maximum growth rates. Nevertheless, the allometries established in this study on life history traits are still only hypotheses to be tested in the future.

## Supporting Information

Table S1 **Mass estimates for preserved growth annuli in sauropodomorph dinosaurs.** The number of preserved growth annuli (No.) for each specimen with the percentage bone length (%) and mass (kg) estimates for each annulus are shown. For more information, please refer to Table 1 and Figure 1.
(DOC)

## References

[1] West GB, Brown JH, Enquist BJ (1997) A general model for the origin of allometric scaling laws in biology. Science 276: 122–126. 9082983 10.1126/science.276.5309.122

[2] Brown JH, Gillooly JF, Allen AP, Savage VM, West GB (2004) Towards a metabolic theory of ecology. Ecology 85: 1771–1789.

[3] Glazier DS (2009) A unifying explanation for diverse metabolic scaling in animals and plants. Biol Rev 84: 111–138. 10.1111/j.1469-185X.2009.00095.x 19895606

[4] Blueweiss L, Fox H, Kudzma V, Nakashima D, Peters R, et al (1978) Relationships between body size and some life history parameters. Oecologia 37: 257–272. 28309655 10.1007/BF00344996

[5] Gould SJ (1971) Geometric similarity in allometric growth: a contribution to the problem of scaling in the evolution of size. Am Nat 105: 113–136.

[6] Calder WA III (1984) Size, function, and life history. Cambridge, Massachusetts: Harvard University Press. 431 p.

[7] Schmidt-Nielsen K (1984) Scaling: why is animal size so important? Cambridge: Cambridge University Press. 241 p.

[8] Klein N, Remes K, Gee CT, Sander M (eds.) (2011) Biology of the Sauropod Dinosaurs: Understanding the life of giants. Life of the Past (series ed. Farlow J) Bloomington: Indiana University Press, 344 p.

[9] Sander PM, Christian A, Clauss M, Fechner R, Gee CT, et al (2011a) Biology of the sauropod dinosaurs: the evolution of gigantism. Biol Rev Camb Philos Soc 86: 117–155. 21251189 10.1111/j.1469-185X.2010.00137.x PMC3045712

[10] Janis CM, Carrano M (1992) Scaling of reproductive turnover in archosaurs and mammals: why are large terrestrial mammals so rare? Acta Zoologica Fennica 28: 201–206.

[11] Sander PM, Clauss M (2008) Sauropod gigantism. Science 322: 200–201. 18845734 10.1126/science.1160904

[12] Griebeler EM, Werner J (2011) The life cycle of sauropod dinosaurs. In: Klein N, Remes K, Gee CT, Sander PM, eds. Biology of the Sauropod Dinosaurs: Understanding the Life of Giants. Bloomington: Indiana University Press. 263–275.

[13] Werner J, Griebeler EM (2011) Reproductive biology and its impact on body size: Comparative analysis of mammalian, avian and dinosaurian reproduction. PLoS ONE 6: e28442. 22194835 10.1371/journal.pone.0028442 PMC3237437

[14] Fitzhugh HA (1976) Analysis of growth curves and strategies for altering their shape. J Anim Sci 42: 1036–1051. 770411 10.2527/jas1976.4241036x

[15] Cooper LN, Lee AH, Taper ML, Horner JR (2008) Relative growth rates of predators and prey dinosaurs reflect effects of predation. Proc R Soc Lond B 275: 2609–2615. 10.1098/rspb.2008.0912 PMC2605812 18682367

[16] Klein N, Sander PM (2008) Ontogenetic stages in the long bone histology of sauropod dinosaurs. Paleobiology 34: 247–263.

[17] Sander PM, Klein N, Stein K, Wings O (2011b) Sauropod bone histology and its implications for sauropod biology. In: Klein N, Remes K, Gee CT, Sander PM, eds. Biology of the Sauropod Dinosaurs: Understanding the Life of Giants. Bloomington: Indiana University Press. 276–302.

[18] Horner JR, de Ricqlés A, Padian K (2000) Long bone histology of the hadrosaurid dinosaur *Maiasaurua pebblesorum*: growth dynamics and physiology based on an ontogenetic series of skeletal elements. J Vert Paleontol 20: 115–129.

[19] Erickson GM, Currie PJ, Inouye BD, Winn AS (2004) Gigantism and comparative life-history parameters of tyrannosaurid dinosaurs. Nature 430: 772–775. 15306807 10.1038/nature02699

[20] Erickson GM (2005) Assessing dinosaur growth patterns: a microscopic revolution. Trends Ecol Evol 20: 677–684. 16701457 10.1016/j.tree.2005.08.012

[21] Chinsamy-Turan A (2005) The microstructure of dinosaur bone: deciphering biology with fine-scale techniques. Baltimore: Johns Hopkins University Press. 216 p.

[22] Woodward HN, Horner JR, Farlow JO (2011) Osteohistological evidence for determinate growth in the American alligator. J Herpetol 45: 339–342.

[23] Chinsamy A (1993) Bone histology and growth trajectory of the prosauropod dinosaur *Massospondylus carinatus* (Owen). Modern Geology 18: 319–329.

[24] Sander PM, Klein N (2005) Developmental plasticity in the life history of a prosauropod dinosaur. Science 310: 1800–1802. 16357257 10.1126/science.1120125

[25] Klein N, Sander PM (2007) Bone histology and growth of the prosauropod *Plateosaurus engelhardti* Meyer, 1837 from the Norian bonebeds of Trossingen (Germany) and Frick (Switzerland). Special Papers in Palaeontology 77: 169–206.

[26] Padian K, Horner JR (2004) Dinosaur physiology. In: Weishampel DB, Dodson P, Osmólska H, eds. The Dinosauria. 2nd edition. Berkeley: University of California Press. 660–671.

[27] Köhler M, Marín-Moratalla N, Jordana X, Aanes R (2012) Seasonal bone growth and physiology in endotherms shed light on dinosaur physiology. Nature 487: 358–361. 22763443 10.1038/nature11264

[28] Klein N, Scheyer T, Tütken T (2009) Skeletochronology and isotopic analysis of an individual specimen of *Alligator mississippiensis* (Crocodylia: Alligatoridae). Fossil Record 12(2): 121–131.

[29] Carrano MT (2006) Body-size evolution in the Dinosauria. In: Carrano MT, Gaudin TJ, Blob RW, Wible JR, eds. Amniote Paleobiology: Perspectives on the Evolution of Mammals, Birds, and Reptiles. Chicago: University of Chicago Press. 225–268.

[30] Bonnan MF (2004) Morphometric analysis of humerus and femur shape in Morrison sauropods: implications for functional morphology and paleobiology. Paleobiology 30: 444–470.

[31] Kilbourne BM, Makovicky PJ (2010) Limb bone allometry during postnatal ontogeny in non-avian dinosaurs. Anat Rec 217: 135–152. 10.1111/j.1469-7580.2010.01253.x PMC2913023 20557400

[32] Erickson GM, Curry Rogers K, Yerby SA (2001) Dinosaurian growth patterns and rapid avian growth rates. Nature 412: 429–433. 11473315 10.1038/35086558

[33] Sander PM, Tückmantel C (2003) Bone lamina thickness, bone apposition rates, and age estimates in sauropod humeri and femora. Paläontologische Zeitschrift 76: 161–172.

[34] Lehman TM, Woodward HN (2008) Modelling growth rates for sauropod dinosaurs. Paleobiology 34: 264–281.

[35] Curry KA (1999) Ontogenetic histology of *Apatosaurus* (Dinosauria: Sauropoda): new insights on growth rates and longevity. J Vert Paleontol 19: 654–665.

[36] de Ricqlès A, Meunier FJ, Castanet J, Francillon-Vieillot H (1991) Comparative microstructure of bone. In: Hall BK, ed. Bone. Vol. 3: Bone Matrix and Bone Specific Products. Boca Raton: CRC Press. 1–78.

[37] Reid REH (1981) Lamellar-zonal bone with zones and annuli in the pelvis of a sauropod dinosaur. Nature 292: 49–51.

[38] Sander PM (2000) Long bone histology of the Tendaguru sauropods: implications for growth and biology. Paleobiology 26: 466–488.

[39] Bybee PJ, Lee AH, Lamm ET (2006) Sizing the Jurassic theropod dinosaur *Allosaurus*: Assessing growth strategy and evolution of ontogenetic scaling of limbs. Journal of Morphology 267: 347–359. 16380967 10.1002/jmor.10406

[40] Lee AH, Werning S (2008) Sexual maturity in growing dinosaurs does not fit reptilian growth models. Proc Natl Acad Sci USA 105: 582–587. 18195356 10.1073/pnas.0708903105 PMC2206579

[41] Erickson GM, Curry Rogers K, Varricchio DK, Norell MA, Xu X (2007) Growth patterns in brooding dinosaurs reveals the timing of sexual maturity in non-avian dinosaurs and the genesis of the avian condition. Biol Lett 3: 558–561. 17638674 10.1098/rsbl.2007.0254 PMC2396186

[42] Erickson GM, Tumanova TA (2000) Growth curve of *Psittacosaurus mongoliensis* Osborn (Ceratopsia: Psittacosauridae) inferred from long bone histology. Zool J Linn Soc 130: 551–566.

[43] Erickson GM, Makovicky PJ, Inouye BD, Chang-Fu Z, Gao K-Q (2009) A life table for *Psittacosaurus lujiatunensis*: initial insights into ornithischian population biology. Anat Rec 292: 1514–1521. 10.1002/ar.20992 19711482

[44] Richards FJ (1959) A flexible growth function for empirical use. J Exp Bot 10: 290–300.

[45] Gaillard J-M, Pontier D, Allaine D, Loison A, Herve J-C, et al (1997) Variation in growth form and precocity at birth in eutherian mammals. Proc R Soc Lond B 264: 859–868. 10.1098/rspb.1997.0120 PMC1688443 9225478

[46] Reiss MJ (1989) The allometry of growth and reproduction. New York: Cambridge University Press. 200 p.

[47] Stearns SC (1999) The evolution of life histories. Oxford: Oxford University Press. 249 p.

[48] Ritz J, Griebeler EM, Huber R, Clauss M (2010) Body size development of captive and free-ranging African spurred tortoises (*Geochelone sulcata*): high plasticity in reptilian growth rates. Herpetological Journal 20: 213–216.

[49] Ricklefs RE (1968) Patterns of growth in birds. Ibis 110: 419–451.

[50] Owen-Smith RN (1988) Megaherbivores: The influence of very large body size on ecology. New York: Cambridge University Press. 388 p.

[51] Shrader AM, Ferreira SM, McElveen ME, Lee PC, Moss CJ, et al (2005) Growth and age determination of African savanna elephants. J Zool 270: 40–48.

[52] Ricklefs RE (1973) Patterns of growth in birds. II. Growth rate and mode of development. Ibis 115: 177–201.

[53] Janensch W (1961) Die Gliedmaßen und Gliedmaßengürtel der Sauropoden der Tendaguru-Schichten. Palaeontographica Abt. A 3: 177–235.

[54] Remes K (2009) Taxonomy of Late Jurassic diplodocid sauropods from Tendaguru (Tanzania). Fossil Record 12: 23–46.

[55] Stein K, Sander PM (2009) Histological core drilling: a less destructive method for studying bone histology. In: Brown MA, Kane JF, Parker WG, eds. Methods in Fossil Preparation: Proceedings of the First Annual Fossil Preparation and Collections Symposium. Available at: http://fossilprep.org/Stein%20and%20Sander%202009.pdf.

[56] Sander PM, Peitz C, Jackson FD, Chiappe LM (2008) Upper Cretaceous titanosaur nesting sites and their implications for sauropod dinosaur reproductive biology. Palaeontographica Abt. A 284: 69–107.

[57] Wings O, Schwarz-Wings D, Fowler DW (2011) New sauropod material from the Late Jurassic part of the Shishugou Formation (Junggar Basin, Xinjiang, NW China). Neues Jahrbuch für Mineralogie, Geologie und Paläontologie 262(2): 129–150.

[58] Mazzetta GV, Christiansen P, Farina RA (2004) Giants and bizarres: body size of some southern South American Cretaceous dinosaurs. Historical Biology 2004: 1–13.

[59] Perry SF, Breuer T, Pajor N (2011) Structure and function of the sauropod respiratory system. In: Klein N, Remes K, Gee, CT, Sander PM, eds. Biology of the Sauropod Dinosaurs: Understanding the Life of Giants. Bloomington: Indiana University Press. 57–79.

[60] McIntosh JS, Brett-Surman MK, Farlow JO (1997) Sauropods. In: Farlow JO, Brett-Surman MK, eds. The Complete Dinosaur. Bloomington: Indiana University Press. 264–290.

[61] Yates AM, Wedel MJ, Bonnan MF (in press) The early evolution of postcranial skeletal pneumaticity in sauropodomorph dinosaurs. Acta Palaeontologica Polonica.

[62] Wings O, Sander PM, Tütken T, Fowler DW, Sun G (2007) Growth and life history of Asia's largest dinosaur. J Vert Paleontol 27: 167A.

[63] von Bertalanffy L (1938) A quantitative theory of organic growth. Human Biology 10: 181–213.

[64] von Bertalanffy L (1957) Quantitative laws in metabolism and growth. Q Rev Biol 32: 217–231. 13485376 10.1086/401873

[65] Halliday TR, Verrell PA (1988) Body size and age in amphibians and reptiles. J Herpetol 22: 253–265.

[66] Shine R, Charnov EL (1992) Patterns of survival, growth and maturation in snakes and lizards. Am Nat 139: 1257–1269.

[67] Frazer NB, Ehrhart LM (1985) Preliminary growth models for Green, *Chelonia mydas*, and Loggerhead, *Caretta caretta*, turtles in the wild. Copeia 1: 73–79.

[68] Magnusson WE, Sanaiotti TM (1995) Growth of Caiman crocodilus in Central Amazonia, Brazil. Copeia 2: 498–501.

[69] Pütter A (1920) Wachstumsähnlichkeiten. Pflügers Archive für Gesamte Physiologie Menschen und Tiere 180: 298–340.

[70] Zullinger EM, Ricklefs RE, Redford KH, Mace GM (1984) Fitting sigmoidal equations to mammalian growth curves. J Mammal 65: 607–636.

[71] Andrews RM (1982) Patterns of growth in reptiles. In: Gans C, Pough FH, eds. Biology of the Reptilia Vol. 13, Physiology D. New York: Academic Press. 273–320.

[72] Hailey A, Coulson IM (1999) The growth pattern of the African tortoise *Geochelone pardalis* and other chelonians. Can J Zool 77: 181–193.

[73] Jackson FD, Varricchio DJ, Jackson RA, Vila B, Chiappe LM (2008) Comparison of water vapor conductance in a titanosaur egg from the Upper Cretaceous of Argentina and a *Megaloolithus siruguei* egg from Spain. Paleobiology 34: 229–246.

[74] Akaike H (1973) Information theory and extension of the maximum likelihood principle. In: Petrov BN, Csaki F, eds. Proceedings of the 2^nd^ International Symposium On Information Theory. Budapest: Akadémiai Kiadó. 276–281.

[75] Burnham KP, Anderson DR (2002) Model selection and multimodal inference: a practical information-theoretic approach. New York: Springer. 488 p.

[76] Horner JR, Padian K, de Ricqlés A (1999) Variation in dinosaur skeletochronology indicators: implications for age assessment. Paleobiology 25: 49–78.

[77] Case TJ (1978) On the evolution and adaptive significance of postnatal growth rates in the terrestrial vertebrates. Q Rev Biol 53: 242–282. 10.1086/410622 362471

[78] Chinsamy A, Hillenius WJ (2004) Physiology of non-avian dinosaurs. In: Weishampel DB, Dodson P, Osmólska H, eds. The Dinosauria. 2nd edition. Berkeley: University of California Press. 643–659.

[79] Laws RM, Parker IS, Johnstone RC (1975) Elephants and their habitats: the ecology of elephants in North Bunyoro, Uganda. Oxford: Clarendon. 388 p.

[80] Hendriks AJ, Mulder C (2008) Scaling of offspring number and mass to plant and animal size: model and meta-analysis. Oecologia 155: 705–716. 18196279 10.1007/s00442-007-0952-3 PMC2270366

[81] Del Hoyo Jd, Elliott A, Sargatal J, editors. (1992) Handbook of the birds of the world – volume 1 ostrich to ducks. Barcelona: Lynx Editions. 696 p.

