# Supplementary material for: Aging, Maturation and Growth of Sauropodomorph Dinosaurs as Deduced from Growth Curves Using Long Bone Histological Data: An Assessment of Methodological Constraints and Solutions
Source: PLoS One. 2013 Jun 19;8(6):e67012. doi: 10.1371/journal.pone.0067012 (PMC3686781; doi:10.1371/journal.pone.0067012)
Supplement: Table S1 — Mass estimates for preserved growth annuli in sauropodomorph dinosaurs. The number of preserved growth annuli (No.) for each specimen with the percentage bone length (%) and mass (kg) estimates for each annulus are shown. For more information, please refer to Table 1 and Figure 1. (DOC) [file pone.0067012.s001.doc]

**Table S1**. **Mass estimates for preserved growth annuli in sauropodomorph dinosaurs.**

The number of preserved growth annuli (No.) for each specimen with the percentage bone length (%) and mass (kg) estimates for each annulus are shown. For more information on specimens, please refer to Table 1 and Figure 1.

|  | *Plateosaurus*  *engelhardti* | | indet.  mamenchisaurid | | *Apatosaurus* sp. SMA 0014 | | *Apatosaurus* sp. BYU 601-17328 | | indet. diplodicid MfN.R.2625 | | indet. diplodocid MfN.R.NW4 | | *Camarasaurus* sp. | |
| --- | --- | --- | --- | --- | --- | --- | --- | --- | --- | --- | --- | --- | --- | --- |
| No. | % | kg | % | kg | % | kg | % | kg | % | kg | % | kg | % | kg |
| 1 | 71.3 | 576 | 46.5 | 2519 | 28.2 | 453 | 69.6 | 6129 | 64.8 | 1128 | 31.9 | 378 | 81.3 | 7656 |
| 2 | 74.0 | 643 | 60.8 | 5636 | 31.9 | 656 | 76.0 | 7980 | 72.4 | 1573 | 38.5 | 664 | 88.7 | 9942 |
| 3 | 76.4 | 710 | 67.6 | 7756 | 33.7 | 773 | 81.8 | 9950 | 77.0 | 1892 | 44.4 | 1018 | 89.9 | 10351 |
| 4 | 84.7 | 964 | 70.3 | 8699 | 46.2 | 1993 | 85.7 | 11442 | 80.5 | 2162 | 61.9 | 2759 | 93.3 | 11571 |
| 5 | 88.7 | 1107 | 73.4 | 9897 | 59.1 | 4171 | 90.6 | 13519 | 85.8 | 2617 | 66.3 | 3390 | 98.2 | 13491 |
| 6 | 93.1 | 1280 | 76.0 | 11003 | 62.0 | 4816 | 91.0 | 13698 | 91.4 | 3164 | 70.8 | 4128 | 98.6 | 13657 |
| 7 | 95.2 | 1369 | 78.4 | 12089 | 65.6 | 5704 | 93.7 | 14954 | 95.4 | 3598 | 73.5 | 4619 | 98.9 | 13782 |
| 8 | 96.3 | 1418 | 80.6 | 13136 | 72.9 | 7828 | 94.0 | 15098 | 98.1 | 3913 | 74.7 | 4849 | 99.6 | 14077 |
| 9 | 97.6 | 1474 | 83.3 | 14469 | 74.9 | 8490 | 96.6 | 16386 | **100.0** | **4144** | 75.9 | 5086 | **100.0** | **14247** |
| 10 | 99.0 | 1540 | 85.7 | 15768 | 77.1 | 9261 | 97.2 | 16693 |  |  | 78.2 | 5563 |  |  |
| 11 | 99.7 | 1571 | 88.5 | 17405 | 82.1 | 11182 | 97.7 | 16952 |  |  | 80.5 | 6068 |  |  |
| 12 | **100.0** | **1587** | 90.7 | 18737 | 85.7 | 12718 | 98.1 | 17161 |  |  | 84.0 | 6894 |  |  |
| 13 |  |  | 92.9 | 20135 | 87.8 | 13676 | 98.4 | 17319 |  |  | 91.8 | 8999 |  |  |
| 14 |  |  | 95.8 | 22057 | 89.6 | 14535 | 98.8 | 17531 |  |  | 93.8 | 9600 |  |  |
| 15 |  |  | 97.1 | 22980 | 91.4 | 15428 | 99.1 | 17692 |  |  | 96.1 | 10323 |  |  |
| 16 |  |  | 98.0 | 23613 | 93.4 | 16463 | 99.6 | 17961 |  |  | **100.0** | **11632** |  |  |
| 17 |  |  | 98.3 | 23853 | 95.7 | 17710 | 99.8 | 18069 |  |  |  |  |  |  |
| 18 |  |  | 98.7 | 24094 | 97.1 | 18499 | **100.0** | **18178** |  |  |  |  |  |  |
| 19 |  |  | 99.0 | 24337 | 98.7 | 19428 |  |  |  |  |  |  |  |  |
| 20 |  |  | 99.3 | 24581 | **100.0** | **20206** |  |  |  |  |  |  |  |  |
| 21 |  |  | 99.7 | 24827 |  |  |  |  |  |  |  |  |  |  |
| 22 |  |  | **100.0** | **25075** |  |  |  |  |  |  |  |  |  |  |
